# Supplementary material for: Benchmarking of analysis strategies for data-independent acquisition proteomics using a large-scale dataset comprising inter-patient heterogeneity
Source: Nat Commun. 2022 May 12;13:2622. doi: 10.1038/s41467-022-30094-0 (PMC9098472; doi:10.1038/s41467-022-30094-0)
Supplement: Supplementary file 1 — Supplementary Information [file 41467_2022_30094_MOESM1_ESM.pdf]

Supplementary Information:

Benchmarking of Analysis Strategies for Data-Independent Acquisition Proteomics Using a Large-Scale Dataset Comprising Inter-Patient Heterogeneity

O. Schilling et al

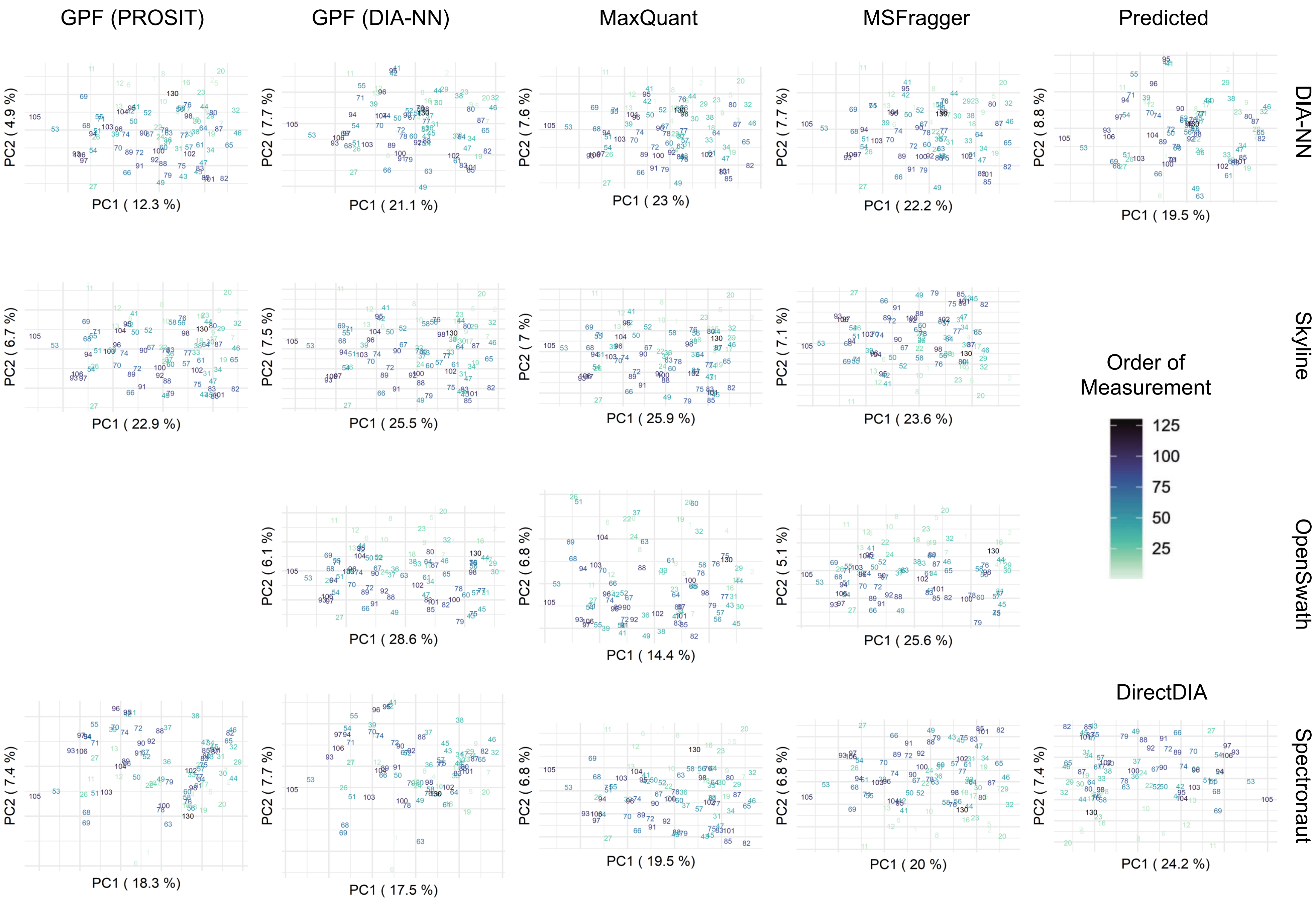

GPF (PROSIT)
GPF (DIA-NN)
MaxQuant
MSFragger
Predicted
DIA-NN
Skyline
OpenSwath
Spectronaut
DirectDIA
PC2 (4.9 %)
PC1 (12.3 %)
PC2 (7.7 %)
PC1 (21.1 %)
PC2 (7.6 %)
PC1 (23 %)
PC2 (7.7 %)
PC1 (22.2 %)
PC2 (8.8 %)
PC1 (19.5 %)
PC2 (6.7 %)
PC1 (22.9 %)
PC2 (7.5 %)
PC1 (25.5 %)
PC2 (7 %)
PC1 (25.9 %)
PC2 (7.1 %)
PC1 (23.6 %)
PC2 (6.1 %)
PC1 (28.6 %)
PC2 (6.8 %)
PC1 (14.4 %)
PC2 (5.1 %)
PC1 (25.6 %)
PC2 (7.4 %)
PC1 (18.3 %)
PC2 (7.7 %)
PC1 (17.5 %)
PC2 (6.8 %)
PC1 (19.5 %)
PC2 (6.8 %)
PC1 (20 %)
PC2 (7.4 %)
PC1 (24.2 %)
Order of Measurement
125
100
75
50
25

Supplementary Figure 1. No Batch Effects Are Observed in this Benchmark Dataset.

NIPALS (Nonlinear Iterative Partial Least Squares) principal component analysis (PCA) was performed based on protein abundance of DIA analysis workflows following quantile normalization. Chronological order of measurement was in an ascending manner, indicated by the increasing darkness of label color. Sample 28, which belongs to spike-in condition 1:6, is not included in this plot as it represents an outlier due to a high degree of missing values. NIPALS was used, as it can directly be applied to data with missing values. Source data are provided as a Source Data file.

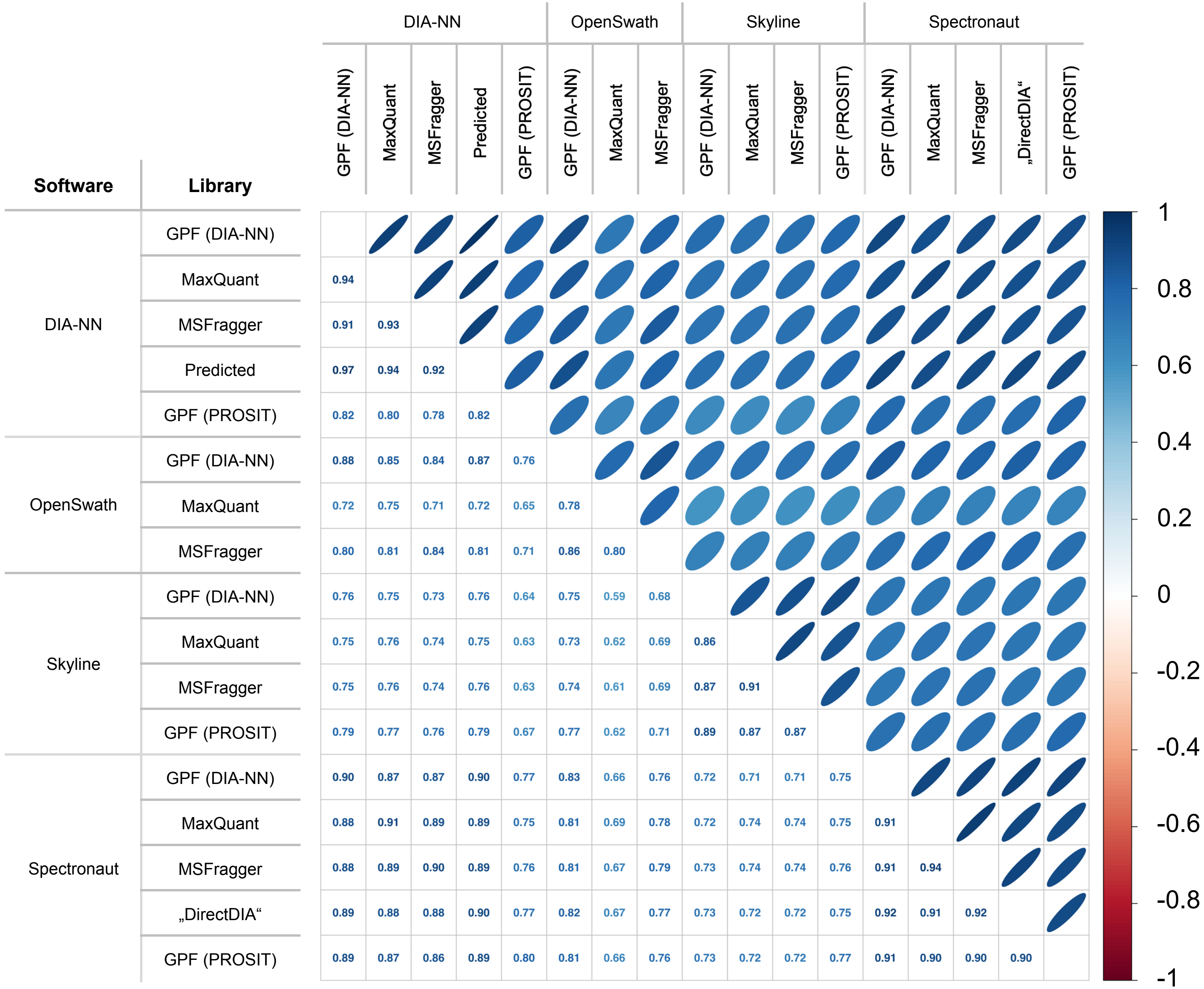

DIA-NN
OpenSwath
Skyline
Spectronaut
Software
Library
GPF (DIA-NN)
MaxQuant
MSFragger
Predicted
GPF (PROSIT)
„DirectDIA“
0.94
0.91 0.93
0.97 0.94 0.92
0.82 0.80 0.78 0.82
0.88 0.85 0.84 0.87 0.76
0.72 0.75 0.71 0.72 0.65 0.78
0.80 0.81 0.84 0.81 0.71 0.86 0.80
0.76 0.75 0.73 0.76 0.64 0.75 0.59 0.68
0.75 0.76 0.74 0.75 0.63 0.73 0.62 0.69 0.86
0.75 0.76 0.74 0.76 0.63 0.74 0.61 0.69 0.87 0.91
0.79 0.77 0.76 0.79 0.67 0.77 0.62 0.71 0.89 0.87 0.87
0.90 0.87 0.87 0.90 0.77 0.83 0.66 0.76 0.72 0.71 0.71 0.75
0.88 0.91 0.89 0.89 0.75 0.81 0.69 0.78 0.72 0.74 0.74 0.75 0.91
0.88 0.89 0.90 0.89 0.76 0.81 0.67 0.79 0.73 0.74 0.74 0.76 0.91 0.94
0.89 0.88 0.88 0.90 0.77 0.82 0.67 0.77 0.73 0.72 0.72 0.75 0.92 0.91 0.92
0.89 0.87 0.86 0.89 0.80 0.81 0.66 0.76 0.73 0.72 0.72 0.77 0.91 0.90 0.90 0.90
1
0.8
0.6
0.4
0.2
0
-0.2
-0.4
-0.6
-0.8
-1

Supplementary Figure 2. Correlation of Protein Intensities Mainly Depends on Employed DIA Analysis Software

Pearson correlation between log2 protein intensities of all DIA analysis workflows (using all complete pairs of observations). The calculated correlation is based on the 3966 proteins common to all DIA analysis workflows. Pearson correlation ranges from -1 (perfect negative correlation, red) to 1 (perfect positive correlation, blue). This is also indicated by the elliptic shape (round indicates lower correlation as opposed to a more narrow shape indicating a higher (positive) correlation). Source data are provided as a Source Data file.

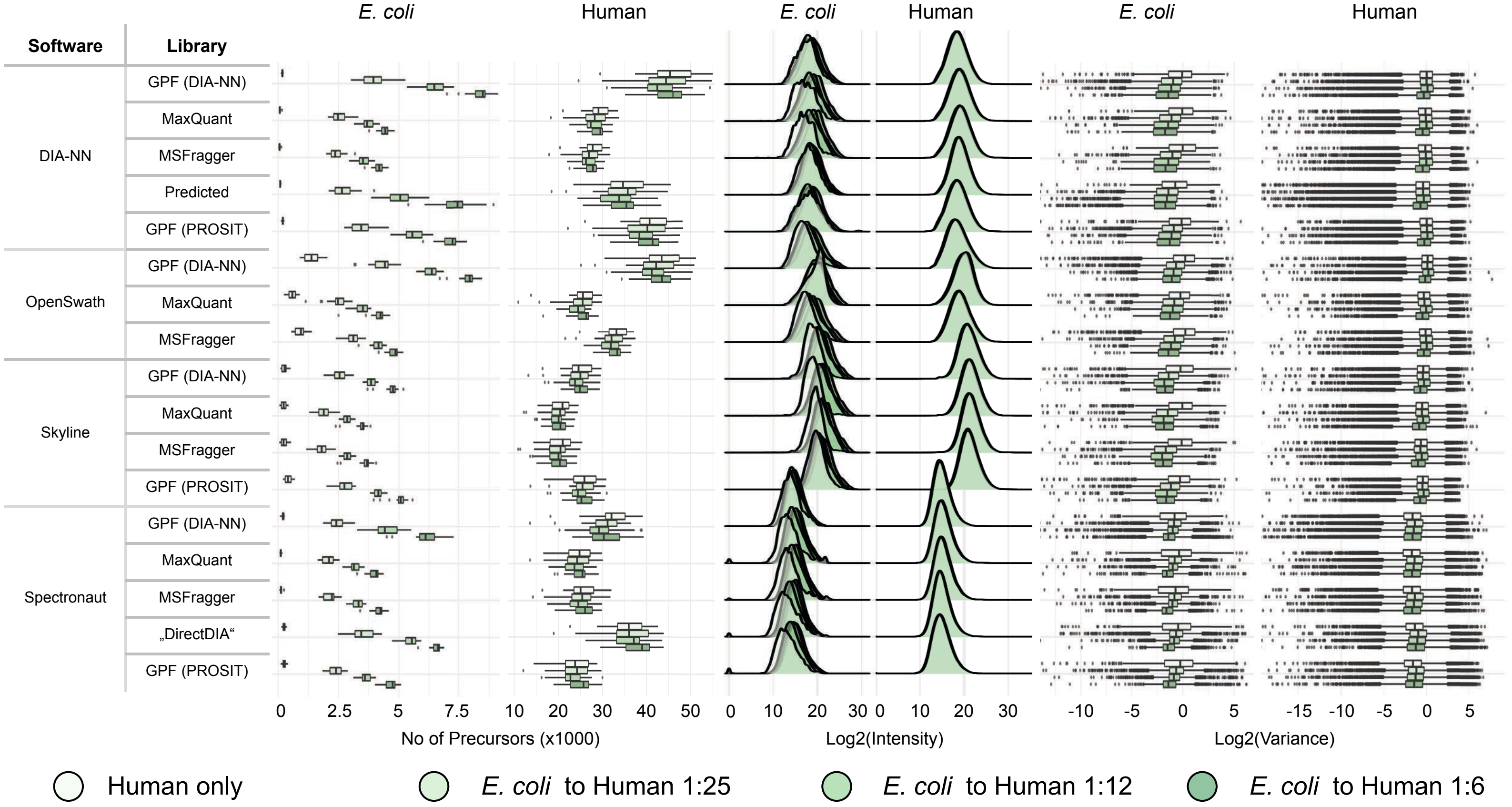

E. coli
Human
E. coli
Human
E. coli
Human
Software
Library
DIA-NN
GPF (DIA-NN)
MaxQuant
MSFragger
Predicted
GPF (PROSIT)
OpenSwath
GPF (DIA-NN)
MaxQuant
MSFragger
Skyline
GPF (DIA-NN)
MaxQuant
MSFragger
GPF (PROSIT)
Spectronaut
GPF (DIA-NN)
MaxQuant
MSFragger
„DirectDIA“
GPF (PROSIT)
0 2.5 5 7.5 10 20 30 40 50
No of Precursors (x1000)
0 10 20 30 0 10 20 30
Log2(Intensity)
-10 -5 0 5 -15 -10 -5 0 5
Log2(Variance)
Human only
E. coli to Human 1:25
E. coli to Human 1:12
E. coli to Human 1:6

Supplementary Figure 3: Precursor number, distribution and variance for each DIA analysis workflow separated by species and color-coded by spike-in condition.

Left: Number of all identified and quantified proteins in all 92 samples. Center: Log2 intensity distributions. Intensities smaller than 0 were excluded. Right: Log2 variance. Log2 variance values smaller than -12 were excluded from this plot.
For spike-condition 1:6 data of n=22 biologically independent samples have been used and for each of the other spike-in conditions data of n=23 biologically independent samples have been used. The boxplots show median (center line), interquartile range (IQR, extending from the first to the third quartile) (box), and 1.5 * IQR (whiskers). Source data are provided as a Source Data file.

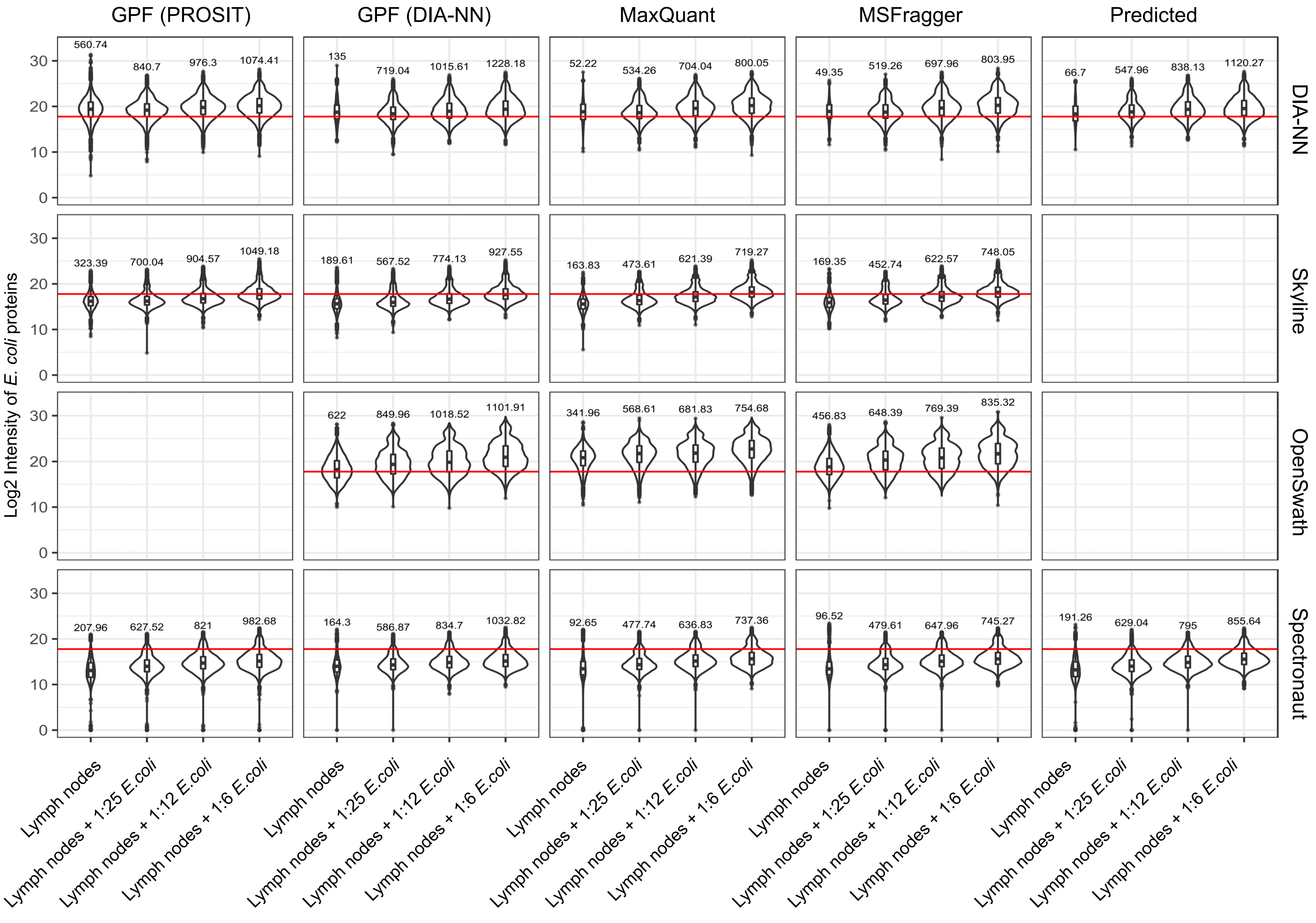

GPF (PROSIT)
GPF (DIA-NN)
MaxQuant
MSFragger
Predicted
DIA-NN
Skyline
OpenSwath
Spectronaut
Log2 Intensity of E. coli proteins
560.74
840.7
976.3
1074.41
135
719.04
1015.61
1228.18
52.22
534.26
704.04
800.05
49.35
519.26
697.96
803.95
66.7
547.96
838.13
1120.27
323.39
700.04
904.57
1049.18
189.61
567.52
774.13
927.55
163.83
473.61
621.39
719.27
169.35
452.74
622.57
748.05
622
849.96
1018.52
1101.91
341.96
568.61
681.83
754.68
456.83
648.39
769.39
835.32
207.96
627.52
821
982.68
164.3
586.87
834.7
1032.82
92.65
477.74
636.83
737.36
96.52
479.61
647.96
745.27
191.26
629.04
795
855.64
Lymph nodes
Lymph nodes + 1:25 E.coli
Lymph nodes + 1:12 E.coli
Lymph nodes + 1:6 E.coli

Supplementary Figure 4: *E. coli* proteins not being present in a biological sample are reported as missing by DIA-NN, Skyline, and Spectronaut, while OpenSwath assumes small intensity values for these missing proteins.

Log2 protein abundance distribution of *E. coli* proteins separated by the four spike-in conditions. The overall median is indicated by the red line. The average number of identified *E. coli* proteins per sample within each DIA analysis workflow is displayed above each violin plot.
For spike-condition 1:6 data of n=22 biologically independent samples have been used and for each of the other spike-in conditions data of n=23 biologically independent samples have been used. The boxplots show median (center line), interquartile range (IQR, extending from the first to the third quartile) (box), and 1.5 * IQR (whiskers). Source data are provided as a Source Data file.

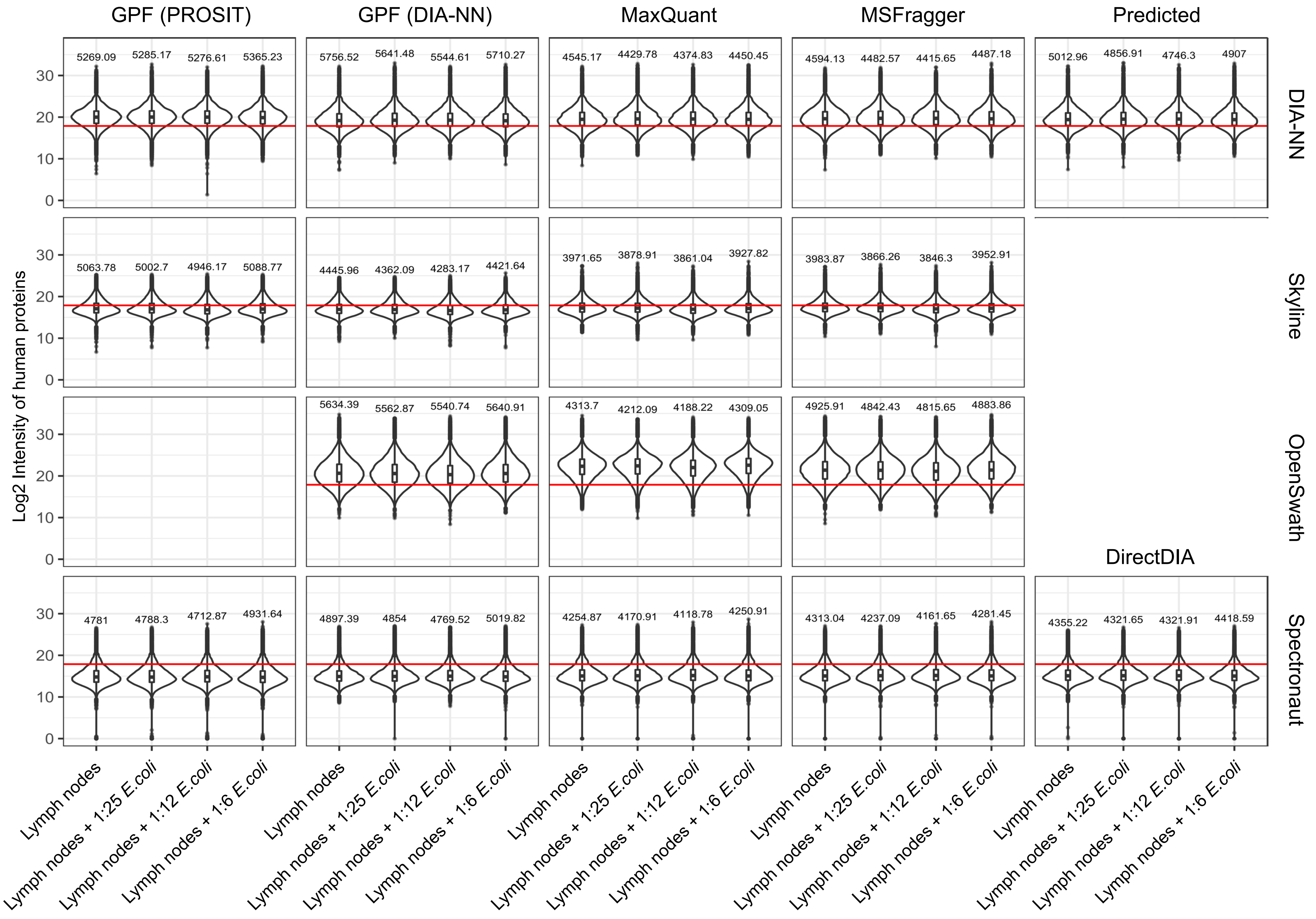

GPF (PROSIT)
GPF (DIA-NN)
MaxQuant
MSFragger
Predicted
DirectDIA
DIA-NN
Skyline
OpenSwath
Spectronaut
Log2 Intensity of human proteins
0
10
20
30
5269.09
5285.17
5276.61
5365.23
5756.52
5641.48
5544.61
5710.27
4545.17
4429.78
4374.83
4450.45
4594.13
4482.57
4415.65
4487.18
5012.96
4856.91
4746.3
4907
5063.78
5002.7
4946.17
5088.77
4445.96
4362.09
4283.17
4421.64
3971.65
3878.91
3861.04
3927.82
3983.87
3866.26
3846.3
3952.91
5634.39
5562.87
5540.74
5640.91
4313.7
4212.09
4188.22
4309.05
4925.91
4842.43
4815.65
4883.86
4781
4788.3
4712.87
4931.64
4897.39
4854
4769.52
5019.82
4254.87
4170.91
4118.78
4250.91
4313.04
4237.09
4161.65
4281.45
4355.22
4321.65
4321.91
4418.59
Lymph nodes
Lymph nodes + 1:25 E.coli
Lymph nodes + 1:12 E.coli
Lymph nodes + 1:6 E.coli

Supplementary Figure 5: Human proteins are identified and quantified equally across all spike-in conditions.

Log2 protein abundance distribution of human proteins separated by the four spike-in conditions. The overall median is indicated by the red line. The average number of identified human proteins per sample within each DIA analysis workflow is displayed above each violin plot. For spike-condition 1:6 data of n=22 biologically independent samples have been used and for each of the other spike-in conditions data of n=23 biologically independent samples have been used. The boxplots show median (center line), interquartile range (IQR, extending from the first to the third quartile) (box), and 1.5 * IQR (whiskers). Source data are provided as a Source Data file.

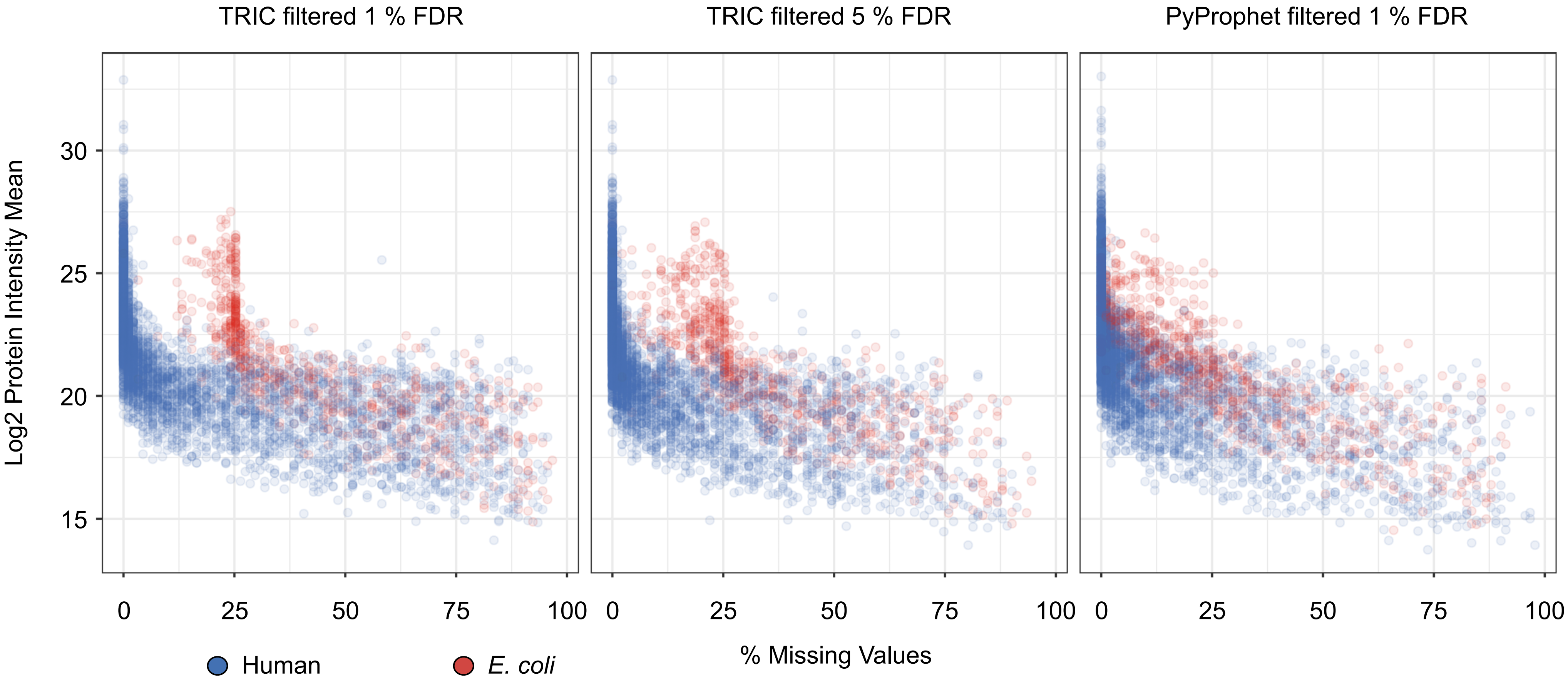

TRIC filtered 1 % FDR
TRIC filtered 5 % FDR
PyProphet filtered 1 % FDR
Log2 Protein Intensity Mean
30
25
20
15
0
25
50
75
100
% Missing Values
Human
E. coli

Supplementary Figure 6. Using TRIC OpenSwath shows a similar missingness behavior

to the other DIA software suites, e.g. for example, the accumulation at 25% missing values, which originates from the human-only samples.
FDR filtering applied to the OpenSwath protein data of the 92 lymph node samples using TRIC with an FDR of 1% (left), TRIC with an FDR of 5% (center), and PyProphet with an FDR of 1 % (right). PyProphet is used in our study. Human and *E. coli* proteins are depicted in blue and red, respectively. Source data are provided as a Source Data file.

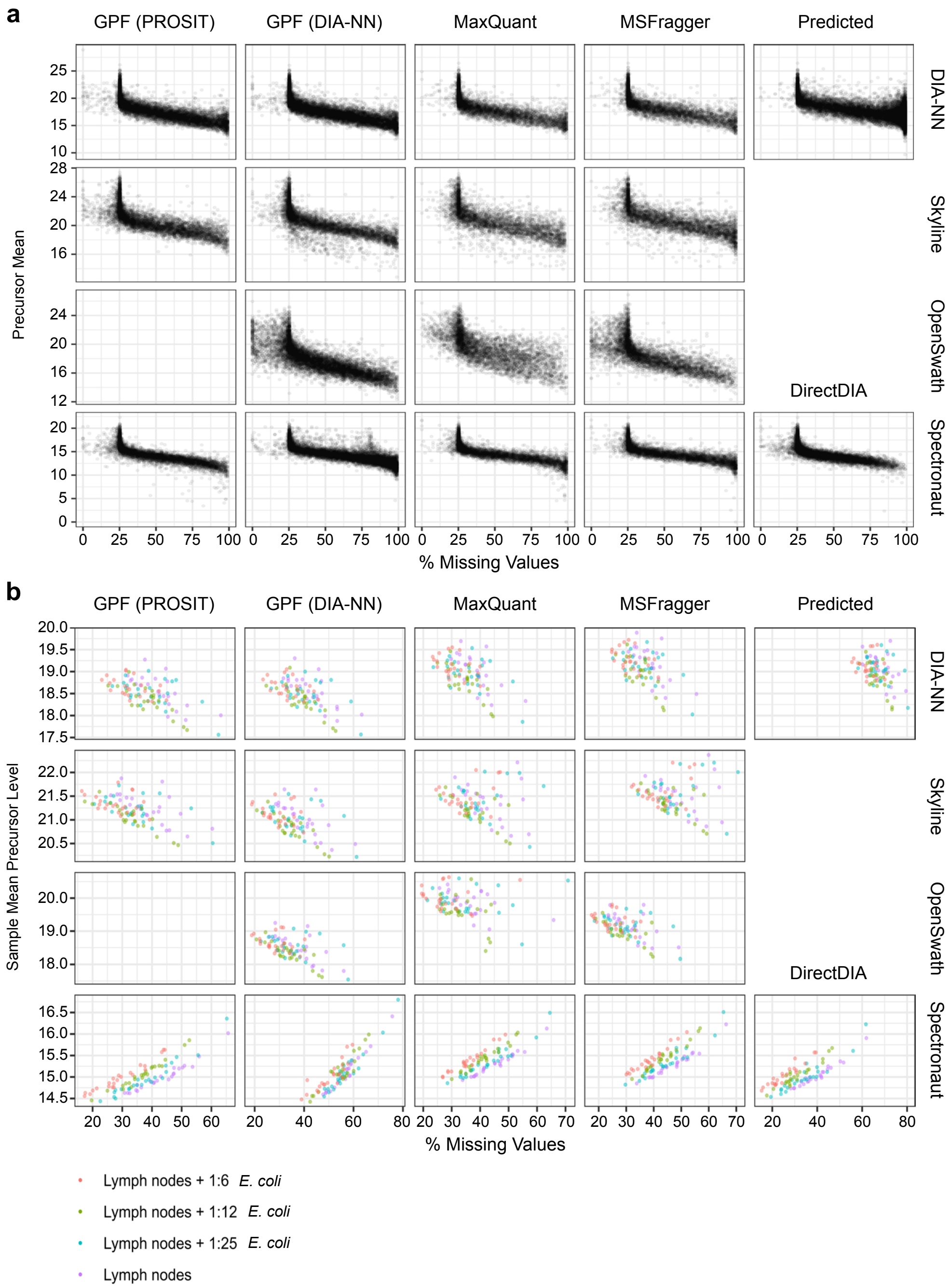
a
GPF (PROSIT)
GPF (DIA-NN)
MaxQuant
MSFragger
Predicted
DIA-NN
Skyline
OpenSwath
DirectDIA
Spectronaut
Precursor Mean
% Missing Values
b
GPF (PROSIT)
GPF (DIA-NN)
MaxQuant
MSFragger
Predicted
DIA-NN
Skyline
OpenSwath
DirectDIA
Spectronaut
Sample Mean Precursor Level
% Missing Values
Lymph nodes + 1:6 E. coli
Lymph nodes + 1:12 E. coli
Lymph nodes + 1:25 E. coli
Lymph nodes

Supplementary Figure 7. Missing Value Characteristics and Correlations on the Precursor Level.

a) Unlike on the protein level (Figure 3A) OpenSwath handles precursors of small intensity similarly to the other DIA software suites. Means of log2 intensities of identified *E. coli* precursors plotted against the percentage of missing values in the respective precursor. *E. coli* precursors are not physically present in 25% of samples. b) The correlation between the missingness within samples and the sample mean over all human and *E. coli* precursors of these samples varies with the employed DIA software. Unlike on the protein level DIA-NN shows a negative correlation between missingness and sample mean. Sample means of precursor intensities are plotted against the percentage of missing values in the respective sample. Source data are provided as a Source Data file.

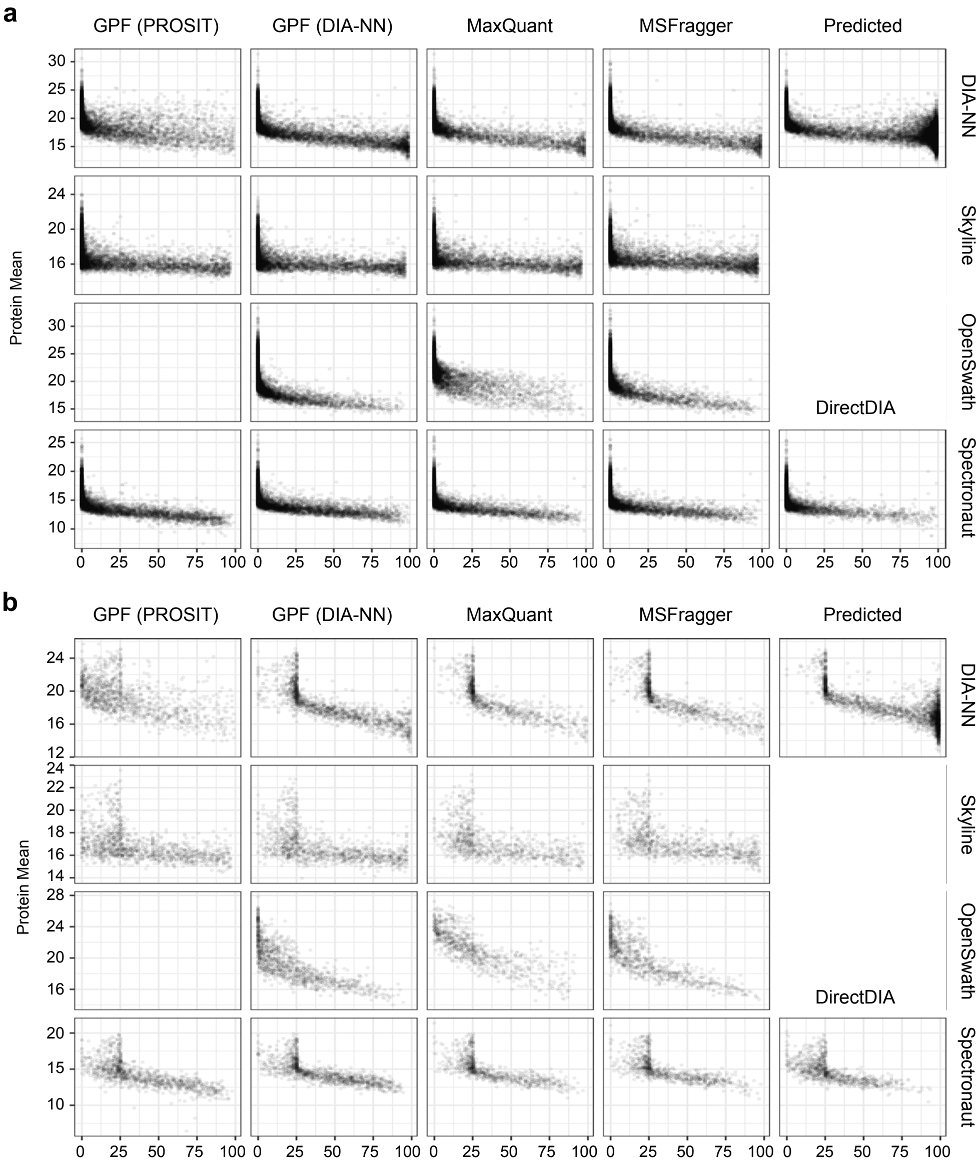
a
GPF (PROSIT)
GPF (DIA-NN)
MaxQuant
MSFragger
Predicted
DIA-NN
Skyline
OpenSwath
Spectronaut
DirectDIA
Protein Mean
30
25
20
15
24
16
10
0
50
75
100
b
GPF (PROSIT)
GPF (DIA-NN)
MaxQuant
MSFragger
Predicted
DIA-NN
Skyline
OpenSwath
Spectronaut
DirectDIA
Protein Mean
12
14
18
22
28

Supplementary Figure 8. Figure 3A separated by species into a) human and b) *E. coli* proteins.

Means of log2 intensities of identified proteins plotted against the percentage of missing values in the respective protein. *E. coli* proteins are not physically present in 25% of samples. Source data are provided as a Source Data file.

True Positive Rate (TPR) = # TP/ **# *E. coli* proteins**

False Positive Rate (FPR) = # FP/ **# Human proteins**

<u>Problem</u>:

No ground truth about number of existing total and regulated proteins in the biological samples known

→ # *E. coli* proteins and # human proteins can refer to different protein lists:

	Protein list	All proteins present in…	Identification quality	Quantification quality	Theoretical maximum of TPR
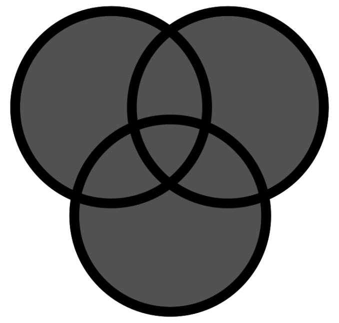	***Combined*** (*E. coli* 2125, human: 11533)	≥ 1 DIA analysis workflow	TPI, FPI ↑ TNI, FNI ↓	↓	DIA workflow-dependent: # identified / # combined
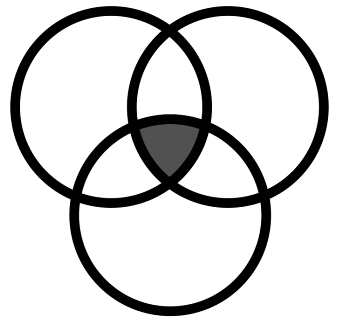	***Intersection*** (*E. coli* 740, Human: 4512)	≥ 80% DIA analysis workflows	TNI, FPI ↓ TNI, FNI ↑	↑	1
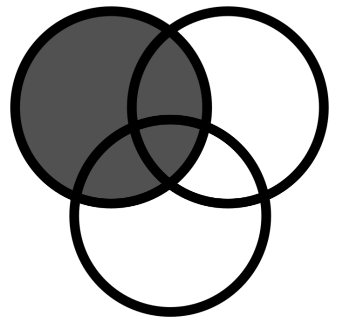	***DIAWorkflow***	DIA workflow-dependent	DIA workflow-dependent	DIA workflow-dependent	1

Supplementary Figure 9. Summary of the Reference Protein Lists.

Abbreviations:
TPI = true positive identifications,
FPI = false positive identifications,
TNI = true negative identifications,
FNI = false negative identifications

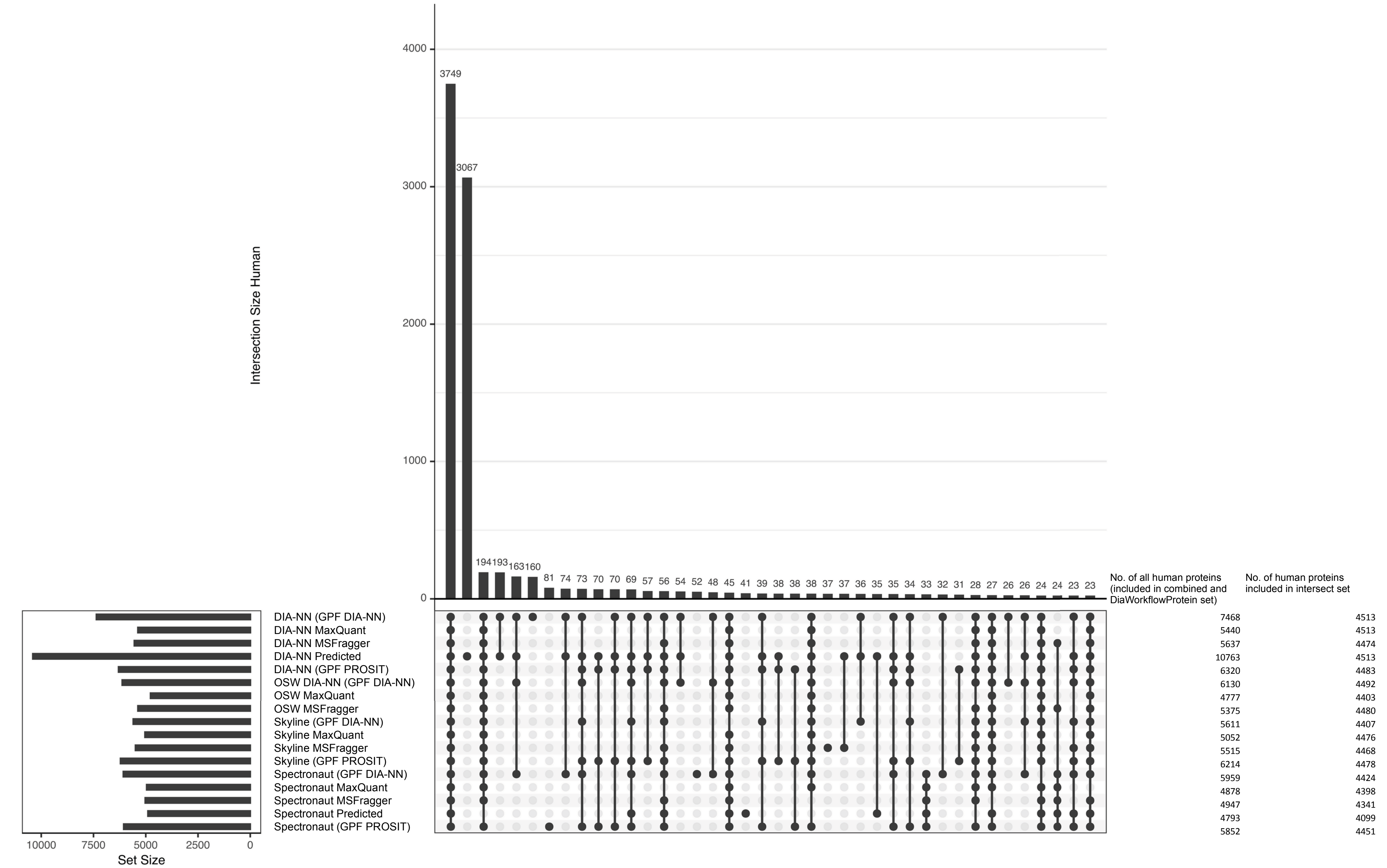
Intersection Size Human
4000
3000
2000
1000
0
3749
3067
194 193 163 160 81 74 73 70 70 69 57 56 54 52 48 45 41 39 38 38 38 37 37 36 35 35 34 33 32 31 28 27 26 26 24 24 23 23
No. of all human proteins (included in combined and DiaWorkflowProtein set)
No. of human proteins included in intersect set
DIA-NN (GPF DIA-NN) 7468 4513
DIA-NN MaxQuant 5440 4513
DIA-NN MSFragger 5637 4474
DIA-NN Predicted 10763 4513
DIA-NN (GPF PROSIT) 6320 4483
OSW DIA-NN (GPF DIA-NN) 6130 4492
OSW MaxQuant 4777 4403
OSW MSFragger 5375 4480
Skyline (GPF DIA-NN) 5611 4407
Skyline MaxQuant 5052 4476
Skyline MSFragger 5515 4468
Skyline (GPF PROSIT) 6214 4478
Spectronaut (GPF DIA-NN) 5959 4424
Spectronaut MaxQuant 4878 4398
Spectronaut MSFragger 4947 4341
Spectronaut Predicted 4793 4099
Spectronaut (GPF PROSIT) 5852 4451
10000 7500 5000 2500 0
Set Size

Supplementary Figure 10: Most Human Protein Identifications Are Shared Between All DIA Workflows with ‘DIA-NN Predicted’ representing the DIA Analysis Workflow capturing the highest Number of Unique Human Proteins.

Overlap of human proteins found in the DIA analysis workflows depicted via an upset plot (left) and number of proteins used in the ‘DIA Workflow’/’Combined’ (left table column) and ‘Intersection’ (right table column) reference protein list (right). Only proteins appearing in one of the spike-in conditions 1:12 or 1:25 were included for this figure and only the 40 largest intersections are displayed in the upset plot. If DIA workflow results contained protein identifiers composed of more than one protein, those proteins are counted as individual entities. In cases where the protein identifier contains more than one protein, proteins contained in the bootstrap datasets are included in the analysis if one of the proteins the protein identifier is composed of matches an entry in the ‘Intersection’ protein list. Unlike for the upset plot, in the table to the right protein identifiers containing multiple protein names are counted as only one protein. Source data are provided as a Source Data file.

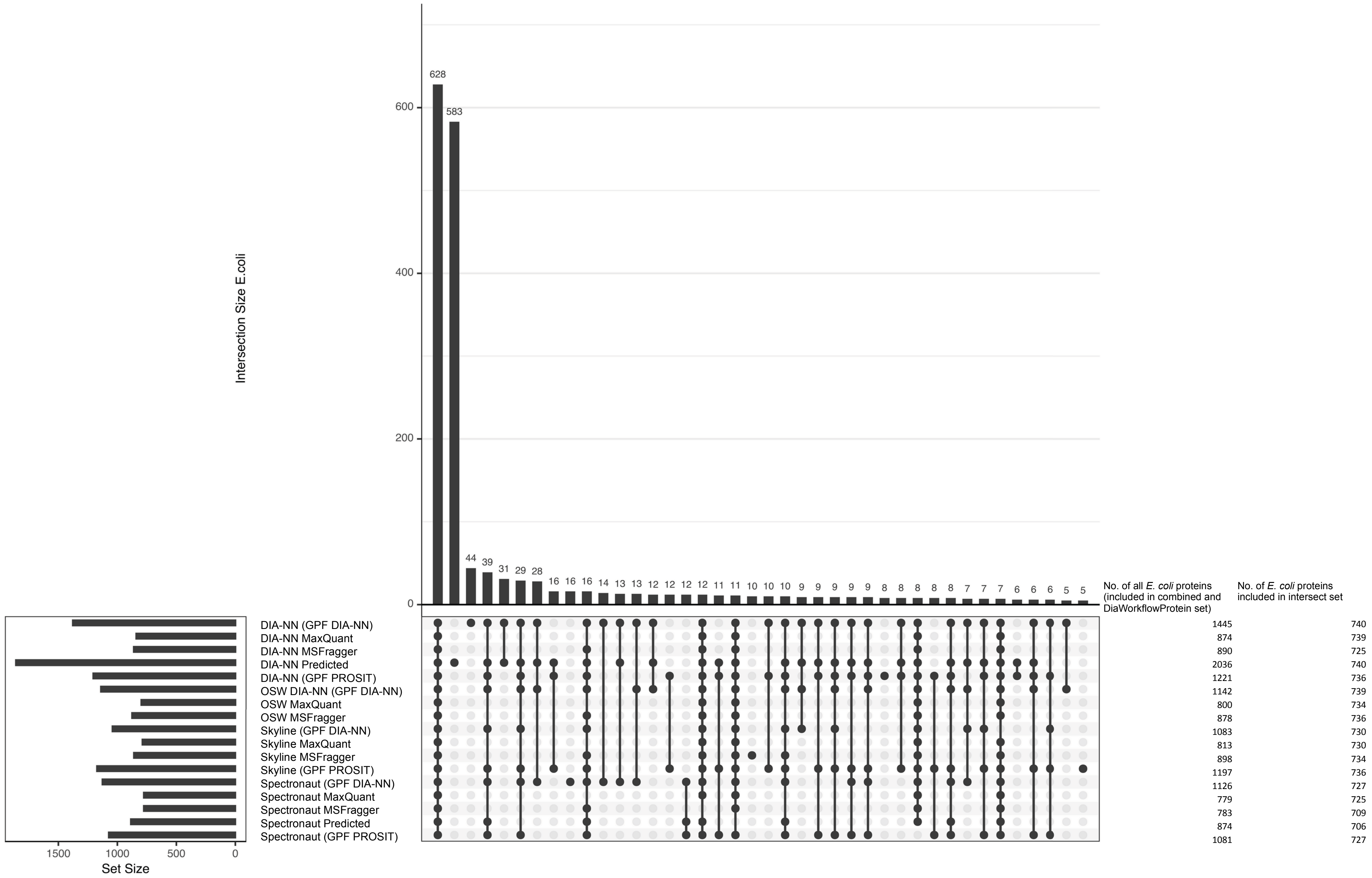
Intersection Size E.coli
628
583
44
39
31
29
28
16
16
16
14
13
13
12
12
12
12
11
11
10
10
10
9
9
9
9
9
8
8
8
8
8
7
7
7
6
6
6
5
5
0
200
400
600
Set Size
1500
1000
500
0
DIA-NN (GPF DIA-NN)
DIA-NN MaxQuant
DIA-NN MSFragger
DIA-NN Predicted
DIA-NN (GPF PROSIT)
OSW DIA-NN (GPF DIA-NN)
OSW MaxQuant
OSW MSFragger
Skyline (GPF DIA-NN)
Skyline MaxQuant
Skyline MSFragger
Skyline (GPF PROSIT)
Spectronaut (GPF DIA-NN)
Spectronaut MaxQuant
Spectronaut MSFragger
Spectronaut Predicted
Spectronaut (GPF PROSIT)
No. of all *E. coli* proteins (included in combined and DiaWorkflowProtein set)
No. of *E. coli* proteins included in intersect set
1445 740
874 739
890 725
2036 740
1221 736
1142 739
800 734
878 736
1083 730
813 730
898 734
1197 736
1126 727
779 725
783 709
874 706
1081 727

Supplementary Figure 11: Most *E. coli* Protein Identifications Are Shared Between All DIA Workflows, with ‘DIA-NN Predicted’ representing the DIA Analysis Workflow capturing the highest Number of Unique *E. coli* Proteins.

See legend of Supplementary Figure 10, but instead of human proteins *E. coli* proteins are summarized. Source data are provided as a Source Data file.

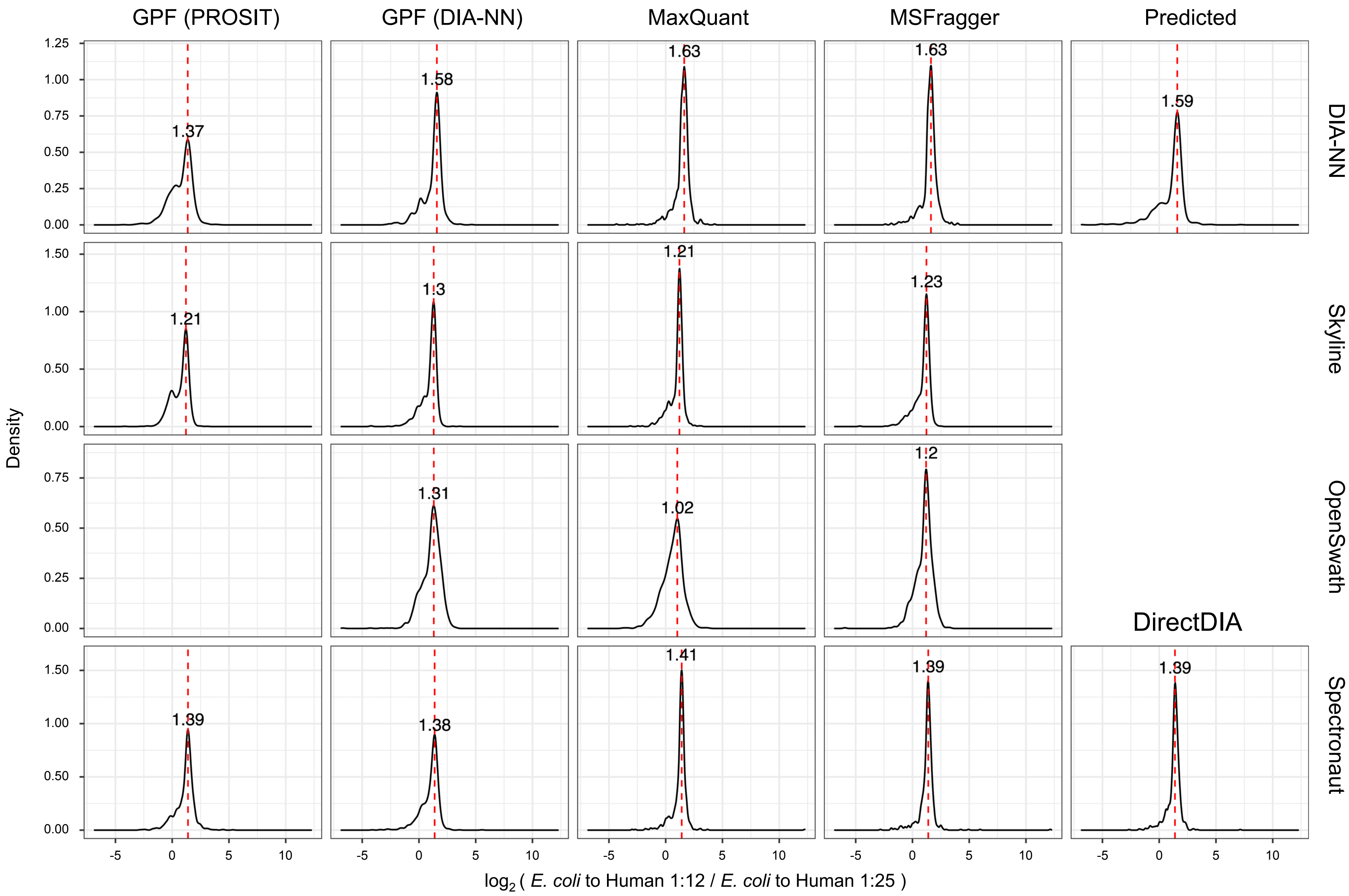

GPF (PROSIT)
GPF (DIA-NN)
MaxQuant
MSFragger
Predicted
DIA-NN
Skyline
OpenSwath
Spectronaut
DirectDIA
Density
1.37
1.58
1.63
1.63
1.59
1.21
1.3
1.21
1.23
1.31
1.02
1.2
1.39
1.38
1.41
1.39
1.39
log2 (E. coli to Human 1:12 / E. coli to Human 1:25)

Supplementary Figure 12: Accuracy of Fold Change Mainly Depends on Employed DIA Analysis Software.

E. coli log2 fold changes observed between the spike-in conditions *E. coli* to human 1:12 and 1:25. The theoretical log2 fold change is 1.11. The dotted red line indicates the mode of the respective distribution. The occurrence of a second smaller peak around 1 likely implies human proteins being incorrectly annotated as *E. coli* proteins. For each spike-in condition data of n=23 biologically independent samples have been used. Source data are provided as a Source Data file.

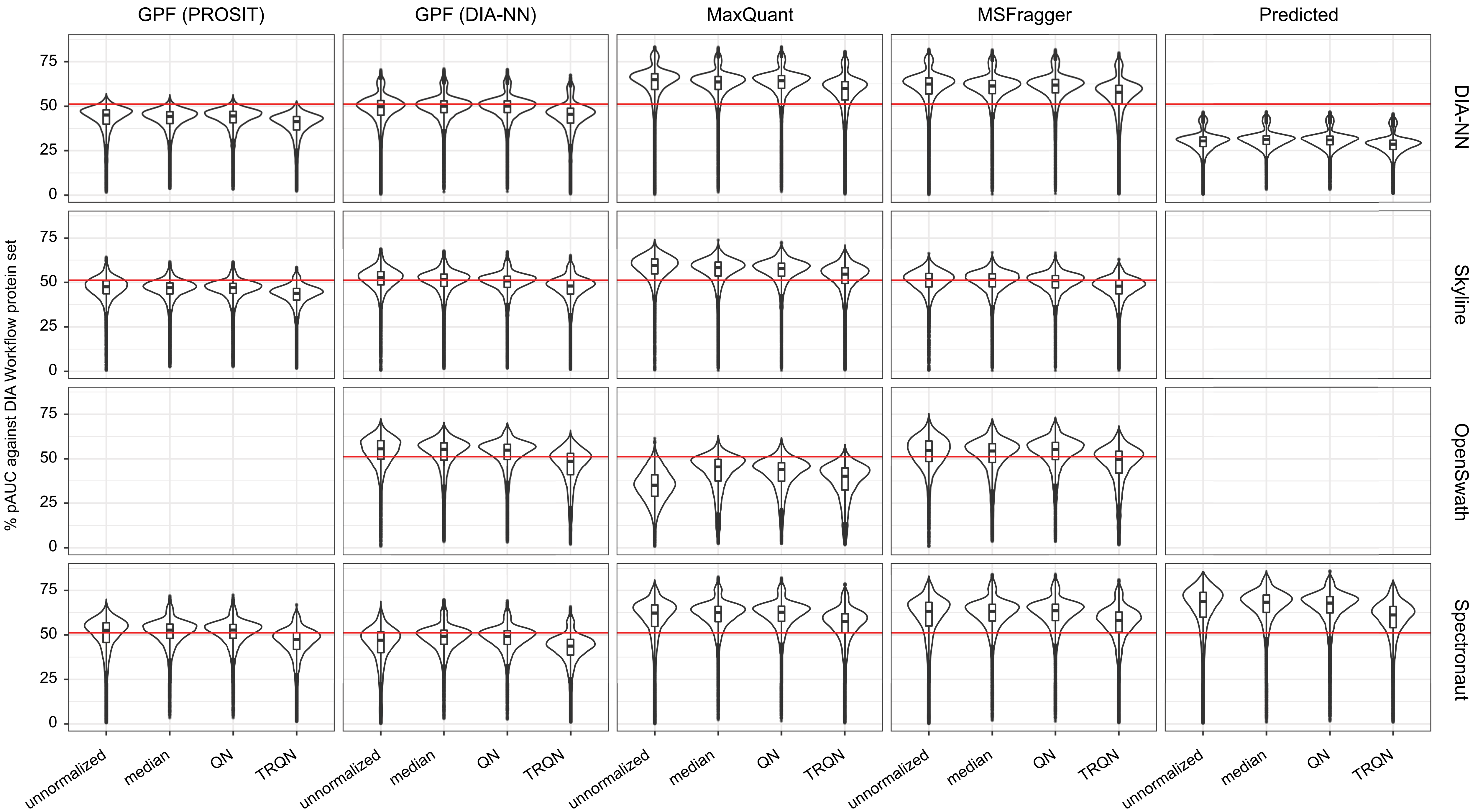

GPF (PROSIT)
GPF (DIA-NN)
MaxQuant
MSFragger
Predicted
DIA-NN
Skyline
OpenSwath
Spectronaut
% pAUC against DIA Workflow protein set
0
25
50
75
unnormalized
median
QN
TRQN

Supplementary Figure 13: Performing no normalization on our benchmark dataset resulted in best prediction performance for most DIA workflows.

Comparison of normalization options for no sparsity reduction (NoSR). pAUC was calculated based on the 'DIA workflow' reference protein list. The red line indicates the overall median. Each subplot is based on n=2100 bootstrap datasets which have been generated by drawing with replacement from data of n=23 biologically independent samples of spike-in conditions 1:12 and 1:25, respectively. The sample size of these bootstrap datasets ranged from 3 to 23 samples, which due to drawing with replacement can appear multiple times. For each subplot that comes to a total of n=2100*3 sparsity reductions*7 statistical tests=44100 data points per normalization setting. The boxplots show median (center line), interquartile range (IQR, extending from the first to the third quartile) (box), and 1.5 * IQR (whiskers). Source data are provided as a Source Data file.

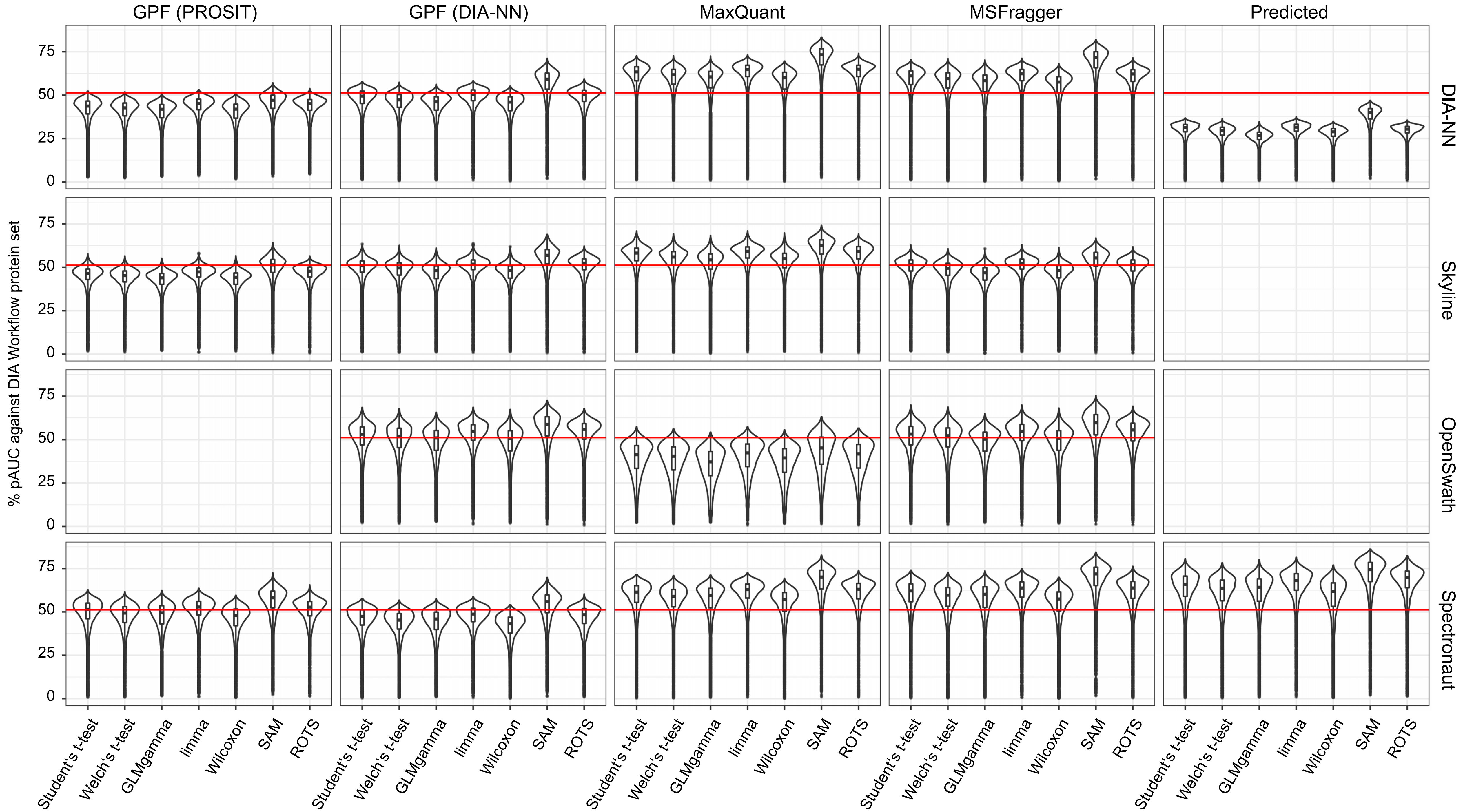

GPF (PROSIT)
GPF (DIA-NN)
MaxQuant
MSFragger
Predicted
DIA-NN
Skyline
OpenSwath
Spectronaut
% pAUC against DIA Workflow protein set
0
25
50
75
Student's t-test
Welch's t-test
GLMgamma
limma
Wilcoxon
SAM
ROTS

Supplementary Figure 14: The statistical test SAM performs best in detecting differentially abundant proteins.

Comparison of statistical tests for no sparsity reduction (NoSR). pAUC was calculated based on the DIAworkflow reference protein list. The red line indicates the overall median. All seven statistical tests were two-sided and not adjusted for multiple testing. Each subplot is based on n=2100 bootstrap datasets which have been generated by drawing with replacement from data of n=23 biologically independent samples of spike-in conditions 1:12 and 1:25, respectively. The sample size of these bootstrap datasets ranged from 3 to 23 samples, which due to drawing with replacement can appear multiple times. For each subplot that comes to a total of n=2100*3 sparsity reductions*4 normalizations=25200 data points per statistical test setting. The boxplots show median (center line), interquartile range (IQR, extending from the first to the third quartile) (box), and 1.5 * IQR (whiskers). Source data are provided as a Source Data file.

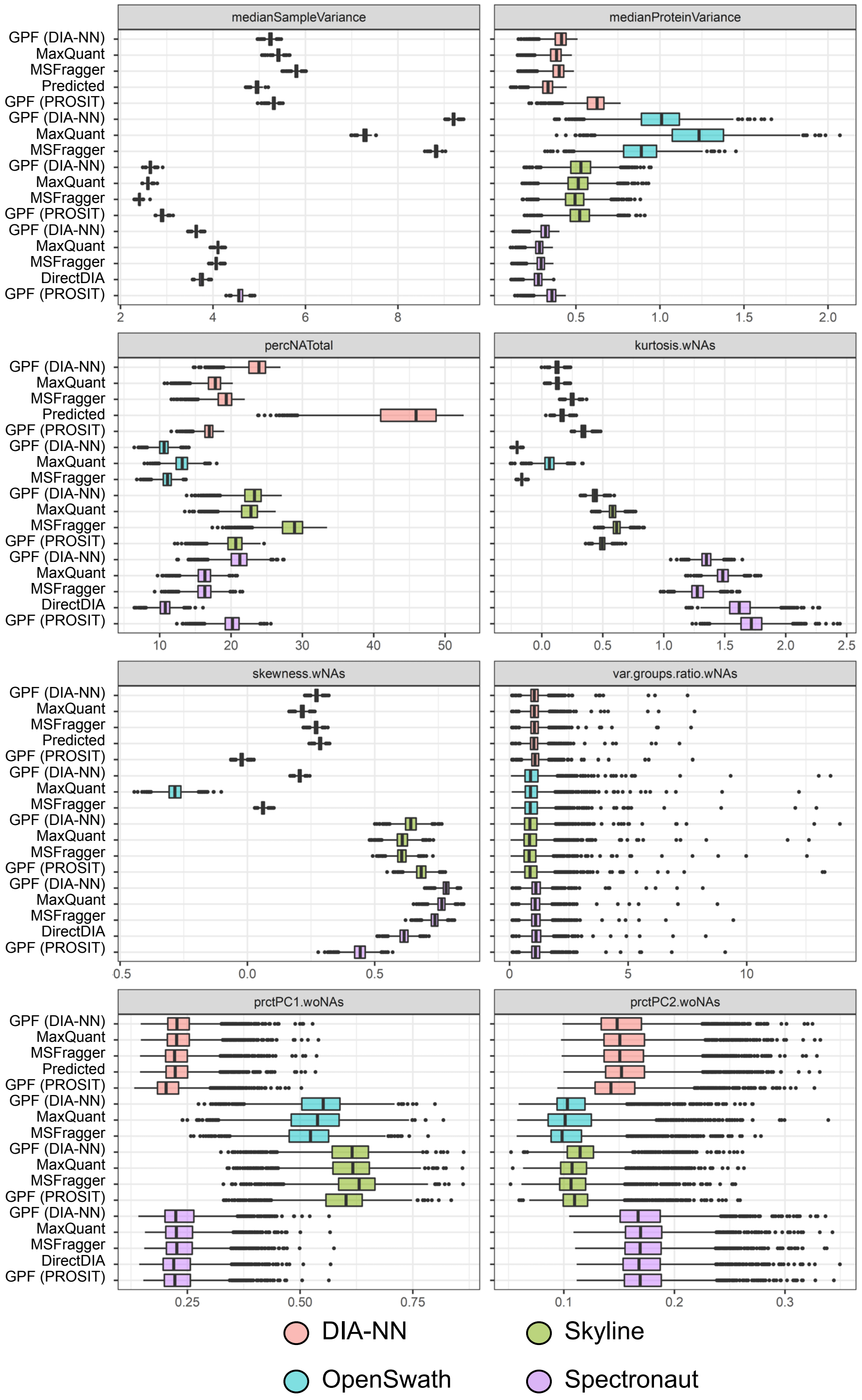

medianSampleVariance
medianProteinVariance
percNATotal
kurtosis.wNAs
skewness.wNAs
var.groups.ratio.wNAs
prctPC1.woNAs
prctPC2.woNAs
GPF (DIA-NN)
MaxQuant
MSFragger
Predicted
GPF (PROSIT)
GPF (DIA-NN)
MaxQuant
MSFragger
GPF (DIA-NN)
MaxQuant
MSFragger
GPF (PROSIT)
GPF (DIA-NN)
MaxQuant
MSFragger
DirectDIA
GPF (PROSIT)
DIA-NN
Skyline
OpenSwath
Spectronaut

Supplementary Figure 15: OpenSwath shows the highest variance in protein intensities (together with Skyline) and sample intensities, as well as the highest percentage of the contribution of the first and second principal component to the total variance.

Distribution of selected data characteristics for all bootstrap datasets of the different DIA analysis workflows. Terminology of data characteristics extensions (.wNAs and .woNAs indicate if proteins were included or excluded if they contained missing values): medianSampleVariance = median of the variances of the samples, medianProteinVariance = median of the variances of the proteins, percNATotal = percentage of missing values, kurtosis.wNAs = kurtosis, skewness.wNAs = skewness, var.groups.ratio.wNAs = median of the ratio of the protein variances of two groups (here: the two spike-in conditions 1:25 and 1:12), prcPC1.woNAs = percentage of the contribution of the first principal component to the total variance, prcPC2.woNAs = percentage of the contribution of the second principal component to the total variance. Each of the eight subplots is based on n=2100 bootstrap datasets which have been generated by drawing with replacement from data of n=23 biologically independent samples of spike-in conditions 1:12 and 1:25, respectively. The sample size of these bootstrap datasets ranged from 3 to 23 samples, which due to drawing with replacement can appear multiple times. The boxplots show median (center line), interquartile range (IQR, extending from the first to the third quartile) (box), and 1.5 * IQR (whiskers). Source data are provided as a Source Data file.

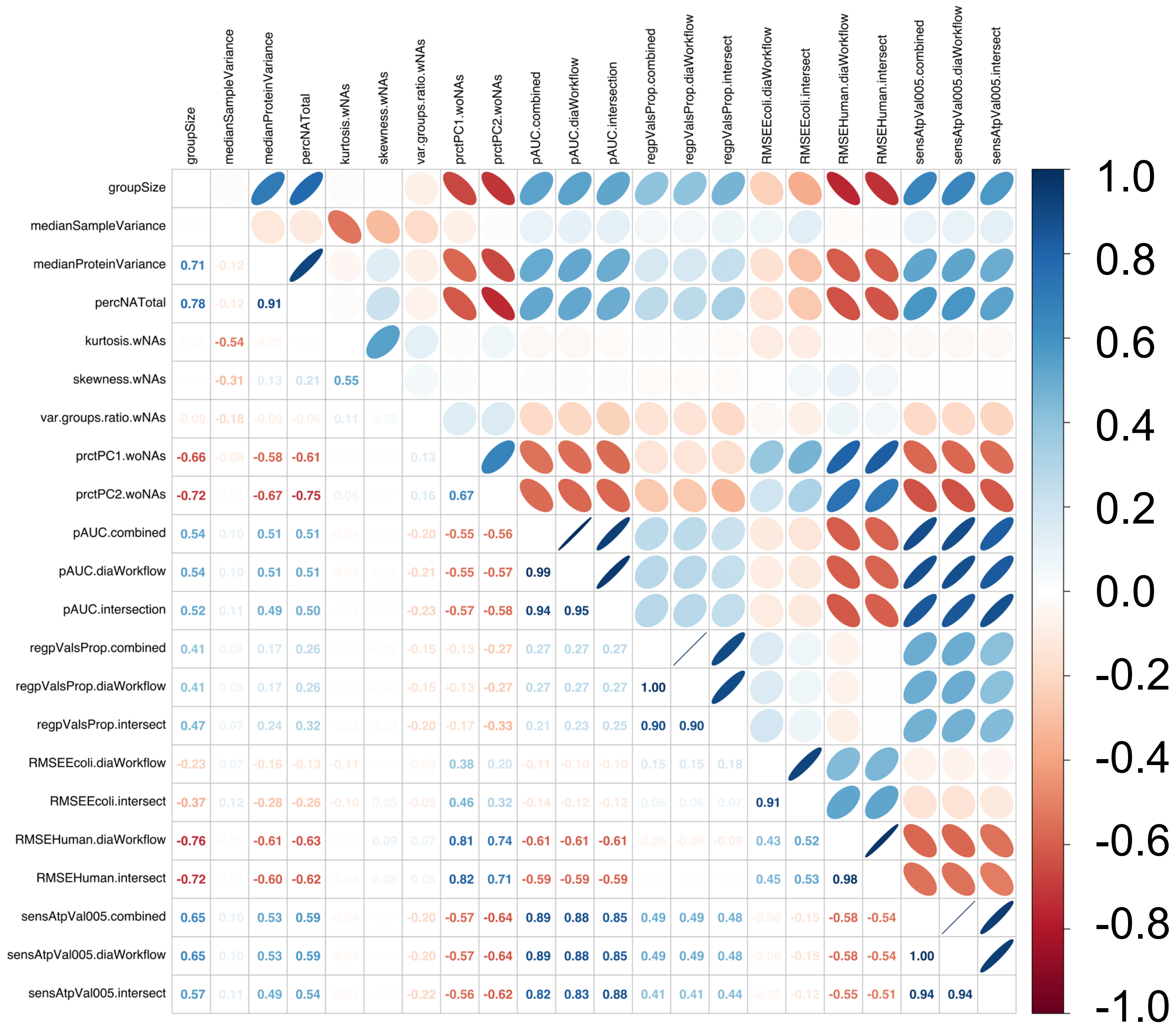
groupSize
medianSampleVariance
medianProteinVariance
percNATotal
kurtosis.wNAs
skewness.wNAs
var.groups.ratio.wNAs
prctPC1.woNAs
prctPC2.woNAs
pAUC.combined
pAUC.diaWorkflow
pAUC.intersection
regpValsProp.combined
regpValsProp.diaWorkflow
regpValsProp.intersect
RMSEEcoli.diaWorkflow
RMSEEcoli.intersect
RMSEHuman.diaWorkflow
RMSEHuman.intersect
sensAtpVal005.combined
sensAtpVal005.diaWorkflow
sensAtpVal005.intersect
1.0
0.8
0.6
0.4
0.2
0.0
-0.2
-0.4
-0.6
-0.8
-1.0

Supplementary Figure 16: Sample variance, kurtosis, skewness and the ratio of variances between two spike-in conditions have little influence on the performance of statistical tests.

Pearson correlations between data characteristics and performance measures of bootstrap datasets (using all complete pairs of observations). The correlations are shown for the DIA analysis workflow that used DIA-NN in combination with the *in silico* predicted GPF-refined (DIA-NN) spectral library. The data characteristics were log2-transformed prior to the correlation analysis. For terminology of data characteristics see Supplementary Figure 15. Terminology of performance measures (extensions .combined, .DiaWorkflow, .intersect/.intersection refer to the reference protein list based on which the performance measure has been calculated): pAUC= partial area under the curve (pAUC), regValsProp = estimated proportion of regulated proteins based on p-value distribution (1-π_0), RMSEEcoli = root-mean-square error (RMSE) of all *E. coli* proteins, RMSEHuman = RMSE of all human proteins, sensAtpVal005 = sensitivity calculated by predicting all proteins with p-value < 0.05 to be differentially abundant. Pearson correlation ranges from -1 (perfect negative correlation, red) to 1 (perfect positive correlation, blue)
The analysis is based on n=2100 bootstrap datasets which have been generated by drawing with replacement from data of n=23 biologically independent samples of spike-in conditions 1:12 and 1:25, respectively. The sample size of these bootstrap datasets ranged from 3 to 23 samples, which due to drawing with replacement can appear multiple times.
That results in a total of n=2100*3 sparsity reductions*4 normalizations*7 statistical tests=176400 cases, for which both prediction performance as well as information on data characteristics are available. Source data are provided as a Source Data file.

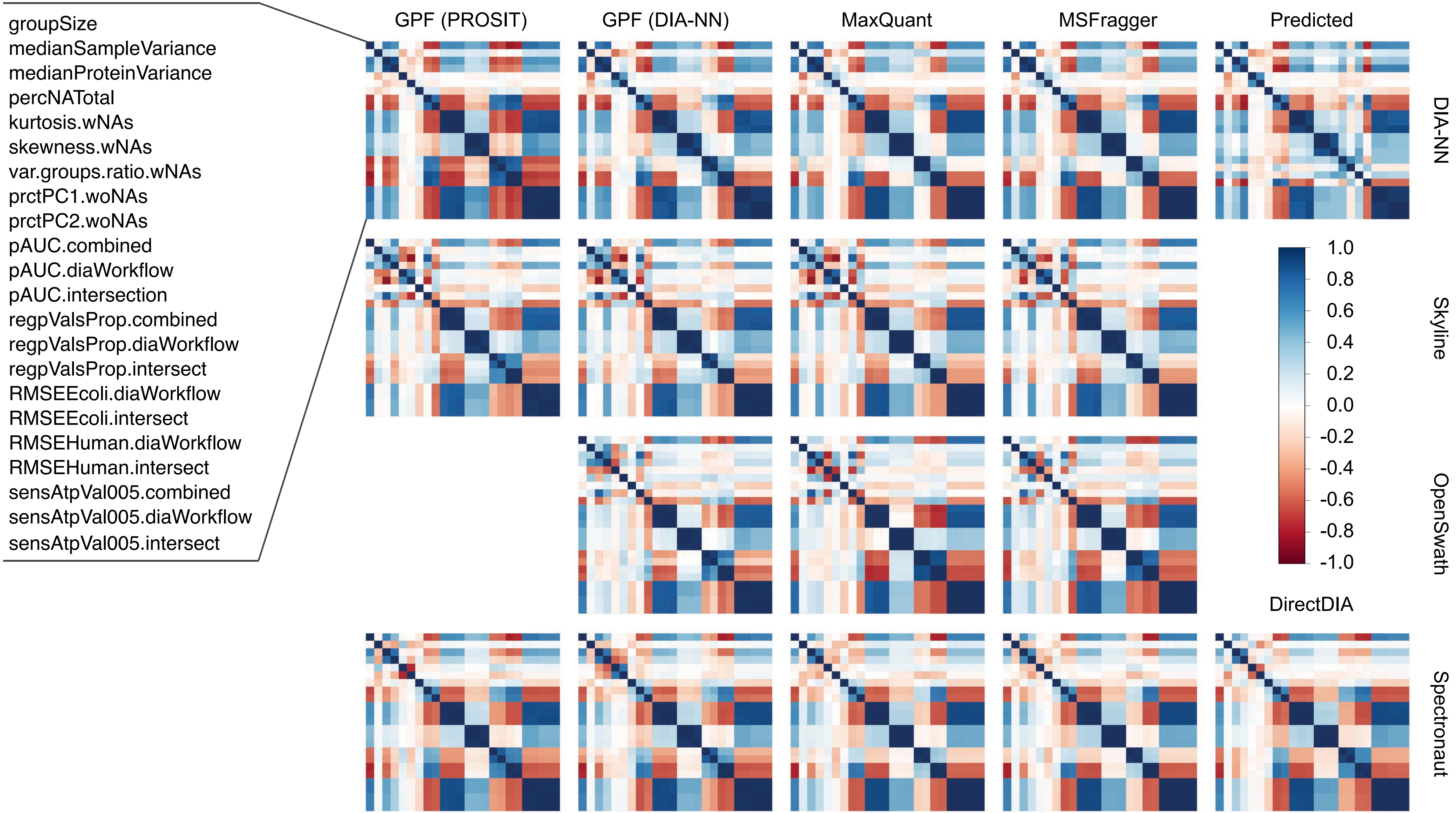

groupSize
medianSampleVariance
medianProteinVariance
percNATotal
kurtosis.wNAs
skewness.wNAs
var.groups.ratio.wNAs
prctPC1.woNAs
prctPC2.woNAs
pAUC.combined
pAUC.diaWorkflow
pAUC.intersection
regpValsProp.combined
regpValsProp.diaWorkflow
regpValsProp.intersect
RMSEEcoli.diaWorkflow
RMSEEcoli.intersect
RMSEHuman.diaWorkflow
RMSEHuman.intersect
sensAtpVal005.combined
sensAtpVal005.diaWorkflow
sensAtpVal005.intersect
GPF (PROSIT)
GPF (DIA-NN)
MaxQuant
MSFragger
Predicted
DIA-NN
Skyline
OpenSwath
Spectronaut
DirectDIA
1.0
0.8
0.6
0.4
0.2
0.0
-0.2
-0.4
-0.6
-0.8
-1.0

Supplementary Figure 17 The correlation between the data characteristics varies with DIA analysis workflow

Pearson correlations between data characteristics and performance measures of bootstrap datasets (using all complete pairs of observations) for all 17 DIA analysis workflows. Pearson correlation ranges from -1 (perfect negative correlation, red) to 1 (perfect positive correlation, blue). The terminology of the data characteristics and performance measures is analogous to Supplementary Figure 16. The analysis is based on n=2100 bootstrap datasets which have been generated by drawing with replacement from data of n=23 biologically independent samples of spike-in conditions 1:12 and 1:25, respectively. The sample size of these bootstrap datasets ranged from 3 to 23 samples, which due to drawing with replacement can appear multiple times. That comes to a total of n=2100*3 sparsity reductions*4 normalizations*7 statistical tests=176400 cases, for which both prediction performance as well as information on data characteristics are available. Source data are provided as a Source Data file.

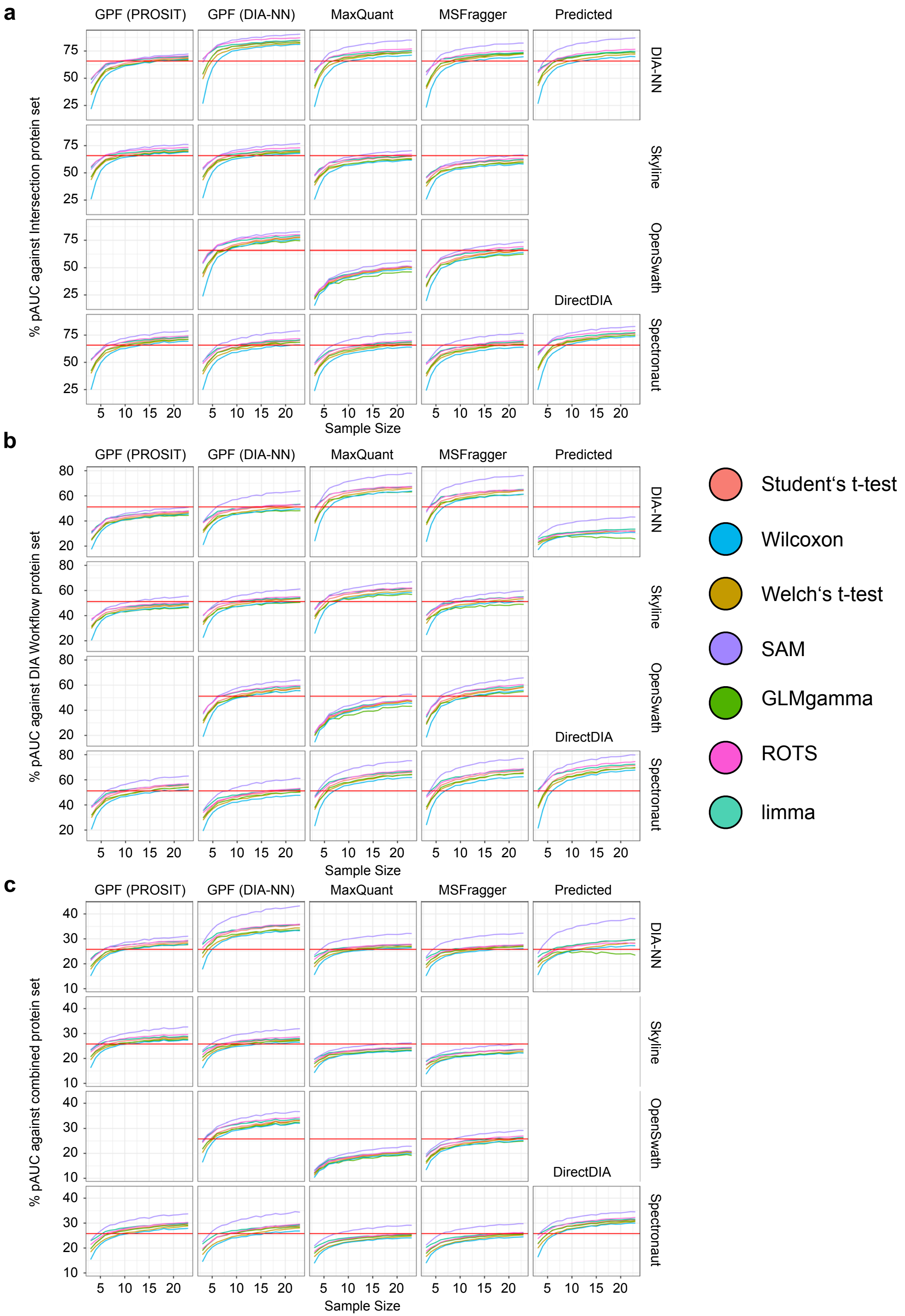

a
GPF (PROSIT)
GPF (DIA-NN)
MaxQuant
MSFragger
Predicted
DIA-NN
Skyline
OpenSwath
DirectDIA
Spectronaut
% pAUC against Intersection protein set
Sample Size
b
% pAUC against DIA Workflow protein set
c
% pAUC against combined protein set
Student's t-test
Wilcoxon
Welch's t-test
SAM
GLMgamma
ROTS
limma

Supplementary Figure 18: SAM performs best for all software suites. limma performs well for small sample sizes.

Prediction performance of different statistical tests in pAUC over different sample sizes for protein reference lists a) Intersection, b) DiaWorkflow, and c) Combined. The lines, which are color-coded by statistical test, represent the median over the respective sample size. All three reference protein lists are similar in terms of the order of best performing statistical tests. The red line indicates the overall median. All seven statistical tests were two-sided and not adjusted for multiple testing.

Each subplot is based on n=2100 bootstrap datasets which have been generated by drawing with replacement from data of n=23 biologically independent samples of spike-in conditions 1:12 and 1:25, respectively. The sample size of these bootstrap datasets ranged from 3 to 23 samples, which due to drawing with replacement can appear multiple times. Each data point the lines are build upon represents the median of n=(2100/21)*3 sparsity reductions*4 normalizations=1200 data points. Source data are provided as a Source Data file.

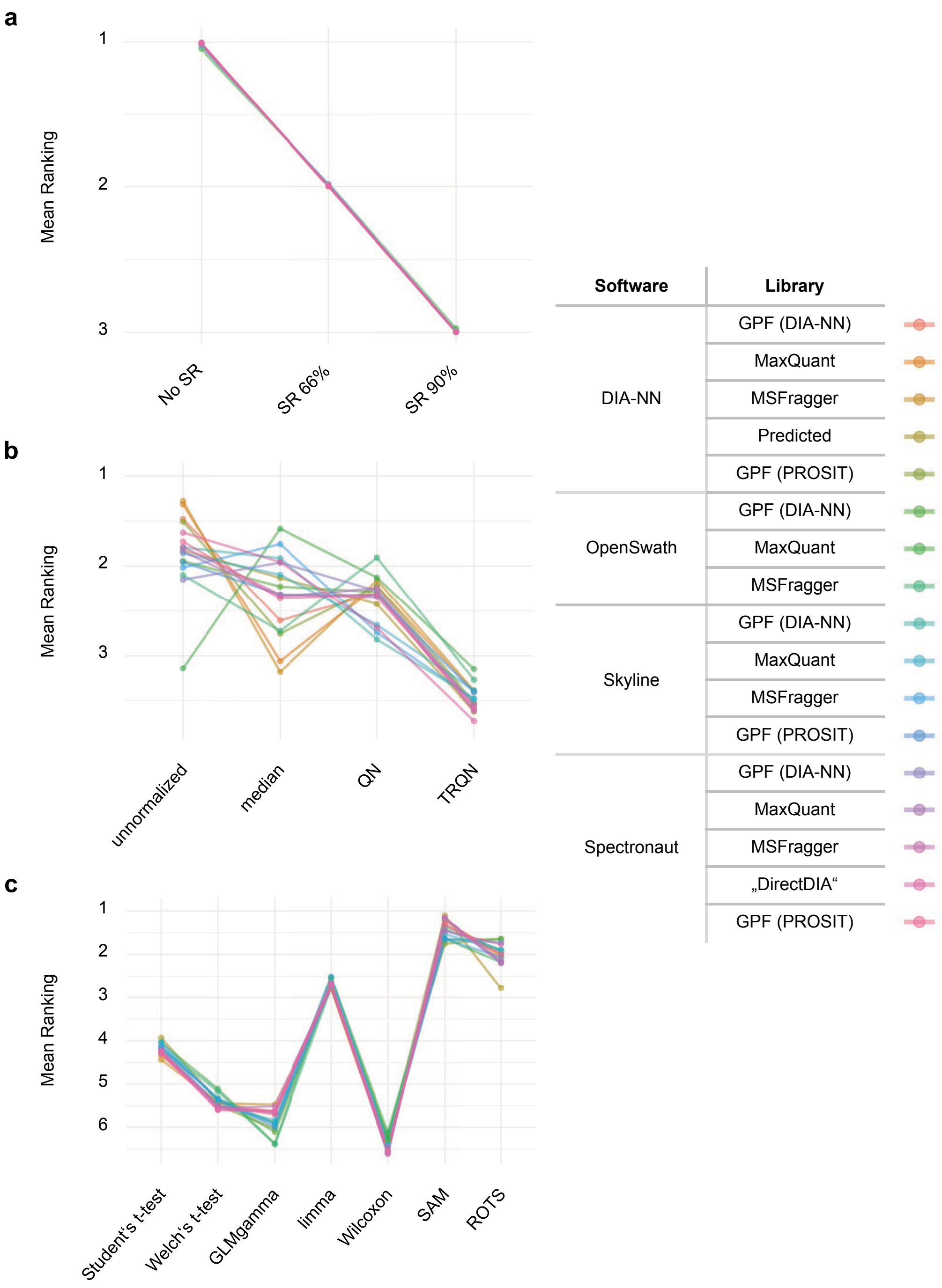

a
Mean Ranking
1
2
3
No SR
SR 66%
SR 90%
b
Mean Ranking
1
2
3
unnormalized
median
QN
TRQN
c
Mean Ranking
1
2
3
4
5
6
Student's t-test
Welch's t-test
GLMgamma
limma
Wilcoxon
SAM
ROTS
Software
Library
DIA-NN
GPF (DIA-NN)
MaxQuant
MSFragger
Predicted
GPF (PROSIT)
OpenSwath
GPF (DIA-NN)
MaxQuant
MSFragger
Skyline
GPF (DIA-NN)
MaxQuant
MSFragger
GPF (PROSIT)
Spectronaut
GPF (DIA-NN)
MaxQuant
MSFragger
„DirectDIA"
GPF (PROSIT)

Supplementary Figure 19: Ranked performance of DIA analysis workflows for different choices of a) sparsity reduction, b) normalization, and c) statistical tests.

Specifically, e.g. for statistical tests, for each bootstrap dataset-DIA workflow-sparsity reduction-normalization-statistical test combination the rank of each statistical test was calculated based on the pAUC. Then the mean rank over all bootstrap dataset-DIA workflow - statistical test combinations was calculated, and, subsequently, the results were averaged over all bootstrap datasets. This has been done for all three reference protein lists and all three results were averaged such that, finally, each statistical test got a rank assigned for each DIA analysis workflow. The lower the rank, the better the performance.
All seven statistical tests were two-sided and not adjusted for multiple testing. Source data are provided as a Source Data file.

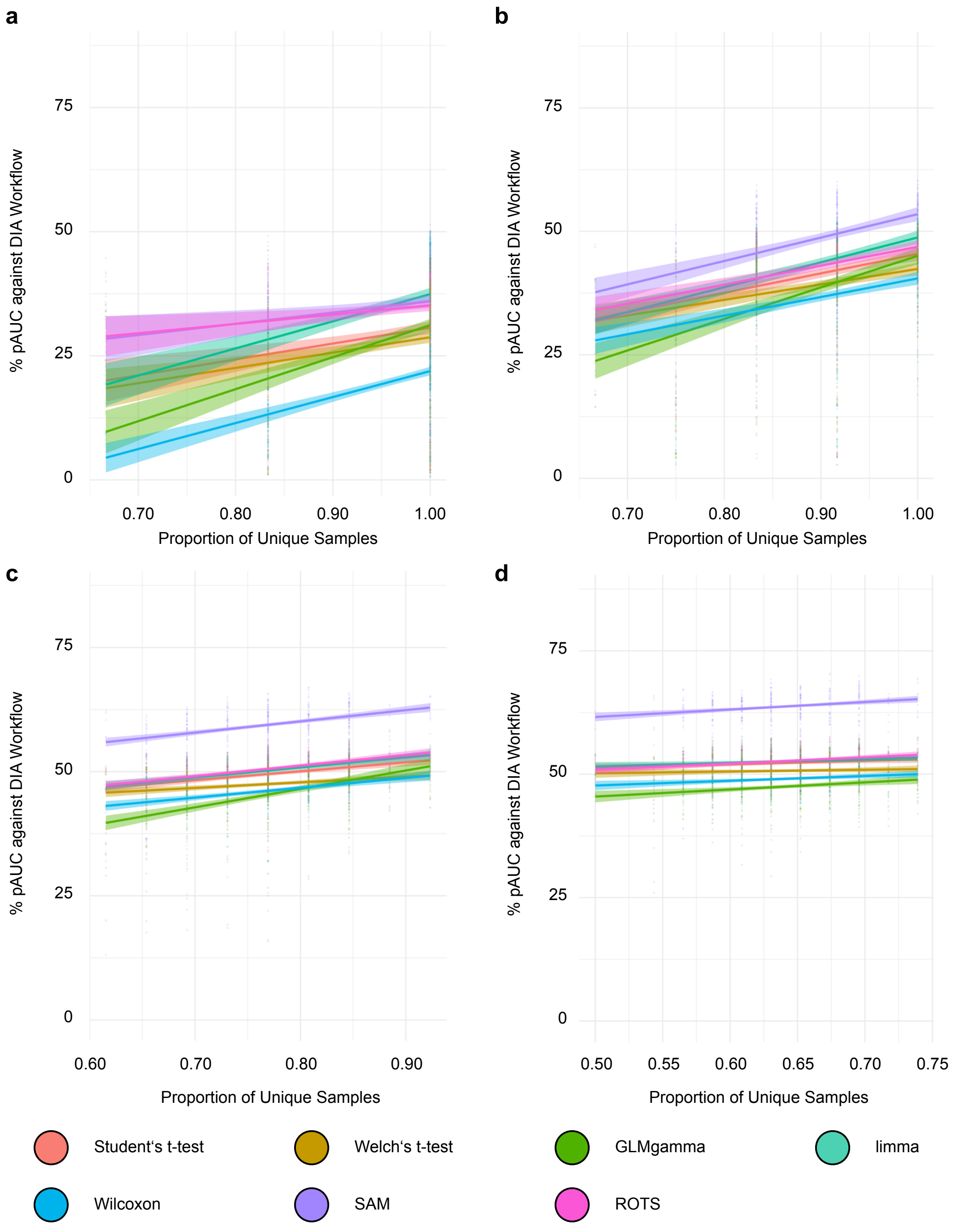
a
b
c
d
% pAUC against DIA Workflow
Proportion of Unique Samples
0
25
50
75
0.70
0.80
0.90
1.00
0.60
0.50
0.55
0.65
0.75
Student's t-test
Welch's t-test
GLMgamma
limma
Wilcoxon
SAM
ROTS

Supplementary Figure 20. All investigated statistical tests show a comparable decrease in prediction performance for an increasing number of duplicate samples in the bootstrap datasets due to bootstrapping with replacement.

This is shown for sample sizes a) 3, b) 6, c) 13, and d) 23 at the example of DIA analysis workflow DIA-NN GPF (DIA-NN) when no sparsity reduction was applied. For each sample size linear regression lines with a 95% confidence interval are depicted for each statistical test. All seven statistical tests were two-sided and not adjusted for multiple testing.
For the comparison of performances it is most important that the bias through duplicated samples is comparable between the different statistical tests, which is, indeed, the case here.
Source data are provided as a Source Data file.
