# Supplementary material for: Benchmarking of analysis strategies for data-independent acquisition proteomics using a large-scale dataset comprising inter-patient heterogeneity
Source: Nat Commun. 2022 May 12;13:2622. doi: 10.1038/s41467-022-30094-0 (PMC9098472; doi:10.1038/s41467-022-30094-0)
Supplement: Supplementary file 3 — Reporting Summary [file 41467_2022_30094_MOESM3_ESM.pdf]

nature portfolio

Corresponding author(s): Oliver Schilling

Last updated by author(s): Mar 22, 2022

Reporting Summary

Nature Portfolio wishes to improve the reproducibility of the work that we publish. This form provides structure for consistency and transparency in reporting. For further information on Nature Portfolio policies, see our Editorial Policies and the Editorial Policy Checklist.

Statistics

For all statistical analyses, confirm that the following items are present in the figure legend, table legend, main text, or Methods section.

n/a	Confirmed	
☐	☒	The exact sample size (*n*) for each experimental group/condition, given as a discrete number and unit of measurement
☒	☐	A statement on whether measurements were taken from distinct samples or whether the same sample was measured repeatedly
☐	☒	The statistical test(s) used AND whether they are one- or two-sided<br>*Only common tests should be described solely by name; describe more complex techniques in the Methods section.*
☒	☐	A description of all covariates tested
☒	☐	A description of any assumptions or corrections, such as tests of normality and adjustment for multiple comparisons
☐	☒	A full description of the statistical parameters including central tendency (e.g. means) or other basic estimates (e.g. regression coefficient) AND variation (e.g. standard deviation) or associated estimates of uncertainty (e.g. confidence intervals)
☐	☒	For null hypothesis testing, the test statistic (e.g. *F*, *t*, *r*) with confidence intervals, effect sizes, degrees of freedom and *P* value noted<br>*Give P values as exact values whenever suitable.*
☒	☐	For Bayesian analysis, information on the choice of priors and Markov chain Monte Carlo settings
☒	☐	For hierarchical and complex designs, identification of the appropriate level for tests and full reporting of outcomes
☐	☒	Estimates of effect sizes (e.g. Cohen's *d*, Pearson's *r*), indicating how they were calculated

Our web collection on statistics for biologists contains articles on many of the points above.

Software and code

Policy information about availability of computer code

Data collection	DIA-NN (1.7.12)<br>Skyline (64 Bit) (20.2.0.343)<br>OpenSwath Workflows (2.6)<br>Spectronaut (14.0)<br>MaxQuant (1.6.14.0)<br>EncyclopeDIA (0.9.5)<br>PROSIT (2019 iRT prediction model)<br>MSFragger (3.2)<br>Fragpipe GUI (14.0)<br>easyPQP (0.1.25)<br>ProteoWizard (3.0.20315)<br>Spectronaut HTRMS converter (14.0)<br>MSstats (3.21.3)<br>PyProphet (2.1.4.2)<br>TRIC (0.11.0)
Data analysis	All code is available under: https://github.com/kreutz-lab/dia-benchmarking (DOI: 10.5281/zenodo.6371925)<br><br>--<br><br>Bootstrapping:

```
R version 4.1.1 (2021-08-10)
Platform: x86_64-apple-darwin17.0 (64-bit)
Running under: macOS Big Sur 10.16

Matrix products: default
LAPACK: /Library/Frameworks/R.framework/Versions/4.1/Resources/lib/libRlapack.dylib

locale:
[1] en_GB.UTF-8/en_GB.UTF-8/en_GB.UTF-8/C/en_GB.UTF-8/en_GB.UTF-8

attached base packages:
[1] stats     graphics  grDevices utils     datasets  methods   base

other attached packages:
[1] dplyr_1.0.7       tidyr_1.1.4       rlist_0.4.6.2     data.table_1.14.2

loaded via a namespace (and not attached):
 [1] zip_2.2.0          Rcpp_1.0.8         pillar_1.6.4       compiler_4.1.1     plyr_1.8.6         viridis_0.6.2
 [7] forcats_0.5.1      tools_4.1.1        corrplot_0.92      viridisLite_0.4.0  lifecycle_1.0.1    tibble_3.1.6
[13] gtable_0.3.0       pkgconfig_2.0.3    rlang_0.4.12       openxlsx_4.2.5     DBI_1.1.2          rstudioapi_0.13
[19] patchwork_1.1.1    parallel_4.1.1     MBQN_2.5.6         gridExtra_2.3      UpSetR_1.4.0       corrr_0.4.3
[25] stringr_1.4.0      generics_0.1.1     vctrs_0.3.8        cowplot_1.1.1      grid_4.1.1         tidyselect_1.1.1
[31] Biobase_2.52.0     glue_1.6.0         R6_2.5.1           fansi_1.0.2        limma_3.48.3       reshape2_1.4.4
[37] purrr_0.3.4        ggplot2_3.3.5      magrittr_2.0.1     BiocGenerics_0.38.0 pcaMethods_1.84.0  scales_1.1.1
[43] ggridges_0.5.3     ellipsis_0.3.2     assertthat_0.2.1   colorspace_2.0-2   utf8_1.2.2         stringi_1.7.6
[49] munsell_0.5.0      crayon_1.4.2

---------------------------------------------------------------------------------

Benchmark analysis:

R version 3.6.3 (2020-02-29)
Platform: x86_64-pc-linux-gnu (64-bit)
Running under: CentOS Linux 7 (Core)

Matrix products: default
BLAS/LAPACK: /gpfs/bwfor/lhome/software/common/compiler/intel/parallel_studio_xe_2019_update4/compilers_and_libraries_2019.4.243/
linux/mkl/lib/intel64_lin/libmkl_intel_lp64.so

locale:
 [1] LC_CTYPE=en_GB.UTF-8       LC_NUMERIC=C
 [3] LC_TIME=en_GB.UTF-8        LC_COLLATE=en_GB.UTF-8
 [5] LC_MONETARY=en_GB.UTF-8    LC_MESSAGES=en_GB.UTF-8
 [7] LC_PAPER=en_GB.UTF-8       LC_NAME=C
 [9] LC_ADDRESS=C               LC_TELEPHONE=C
[11] LC_MEASUREMENT=en_GB.UTF-8 LC_IDENTIFICATION=C

attached base packages:
[1] parallel  stats     graphics  grDevices utils     datasets  methods
[8] base

other attached packages:
 [1] pROC_1.18.0        matrixStats_0.61.0 rlist_0.4.6.2
 [4] pcaMethods_1.78.0  Biobase_2.46.0     BiocGenerics_0.32.0
 [7] doParallel_1.0.15  iterators_1.0.12   foreach_1.5.0
[10] psych_2.1.9        matrixcalc_1.0-5   DescTools_0.99.44
[13] ROTS_1.14.0        samr_3.0           genefilter_1.68.0
[16] qvalue_2.18.0      limma_3.42.2       matrixTests_0.1.9.1
[19] MBQN_1.0.1         dplyr_1.0.7

loaded via a namespace (and not attached):
 [1] colorspace_2.0-2       ellipsis_0.3.2
 [3] class_7.3-15           XVector_0.26.0
 [5] GenomicRanges_1.38.0   fs_1.5.2
 [7] gld_2.6.4              rstudioapi_0.13
 [9] proxy_0.4-26           bit64_4.0.5
[11] AnnotationDbi_1.48.0   fansi_0.5.0
[13] mvtnorm_1.1-3          codetools_0.2-16
[15] splines_3.6.3          mnormt_2.0.2
```

```
[17] cachem_1.0.6            rootSolve_1.8.2.3
[19] impute_1.60.0           jsonlite_1.7.2
[21] annotate_1.64.0         dbplyr_2.1.1
[23] shiny_1.7.1             compiler_3.6.3
[25] httr_1.4.2              assertthat_0.2.1
[27] Matrix_1.2-18           fastmap_1.1.0
[29] later_1.3.0             htmltools_0.5.2
[31] tools_3.6.3             gtable_0.3.0
[33] glue_1.6.0              lmom_2.8
[35] GenomeInfoDbData_1.2.2      reshape2_1.4.4
[37] rappdirs_0.3.3          Rcpp_1.0.7
[39] vctrs_0.3.8             nlme_3.1-144
[41] stringr_1.4.0           openxlsx_4.2.5
[43] mime_0.12               lifecycle_1.0.1
[45] XML_3.99-0.3            zlibbioc_1.32.0
[47] MASS_7.3-51.5           scales_1.1.1
[49] promises_1.2.0.1        SummarizedExperiment_1.16.1
[51] expm_0.999-6            curl_4.3.2
[53] Exact_3.1               memoise_2.0.1
[55] ggplot2_3.3.5           stringi_1.7.6
[57] RSQLite_2.2.9           S4Vectors_0.24.4
[59] e1071_1.7-9             boot_1.3-24
[61] zip_2.2.0               BiocParallel_1.20.1
[63] GSA_1.03.1              GenomeInfoDb_1.22.1
[65] rlang_0.4.12            pkgconfig_2.0.3
[67] bitops_1.0-7            lattice_0.20-38
[69] purrr_0.3.4             bit_4.0.4
[71] tidyselect_1.1.1        plyr_1.8.6
[73] magrittr_2.0.1          R6_2.5.1
[75] IRanges_2.20.2          generics_0.1.1
[77] DelayedArray_0.12.3     DBI_1.1.1
[79] pillar_1.6.4            survival_3.1-8
[81] RCurl_1.98-1.5          tibble_3.1.6
[83] crayon_1.4.2            shinyFiles_0.9.1
[85] utf8_1.2.2              tmvnsim_1.0-2
[87] BiocFileCache_1.10.2    grid_3.6.3
[89] data.table_1.14.2       blob_1.2.2
[91] digest_0.6.29           xtable_1.8-4
[93] httpuv_1.6.4            stats4_3.6.3
[95] munsell_0.5.0

-----------------------------------------------------------------------------

Visualizations:

R version 4.1.1 (2021-08-10)
Platform: x86_64-apple-darwin17.0 (64-bit)
Running under: macOS Monterey 12.2

Matrix products: default
LAPACK: /Library/Frameworks/R.framework/Versions/4.1/Resources/lib/libRlapack.dylib

locale:
[1] en_GB.UTF-8/en_GB.UTF-8/en_GB.UTF-8/C/en_GB.UTF-8/en_GB.UTF-8

attached base packages:
[1] parallel  stats     graphics  grDevices utils     datasets  methods   base

other attached packages:
 [1] ggplotify_0.1.0    doParallel_1.0.17  iterators_1.0.14   foreach_1.5.2      limma_3.48.3
 [6] pcaMethods_1.84.0  Biobase_2.52.0     BiocGenerics_0.38.0 corrr_0.4.3       corrplot_0.92
[11] UpSetR_1.4.0       ggridges_0.5.3     viridis_0.6.2      viridisLite_0.4.0  patchwork_1.1.1
[16] gridExtra_2.3      cowplot_1.1.1      RColorBrewer_1.1-2 ggplot2_3.3.5      reshape2_1.4.4
[21] dplyr_1.0.8        plyr_1.8.6         tidyr_1.2.0        forcats_0.5.1      readr_2.1.2
[26] svglite_2.1.0      openxlsx_4.2.5     rlist_0.4.6.2

loaded via a namespace (and not attached):
 [1] nlme_3.1-155       bitops_1.0-7       bit64_4.0.5        httr_1.4.2
```

[5] GenomeInfoDb_1.28.4	tools_4.1.1	utf8_1.2.2	R6_2.5.1
[9] mgcv_1.8-39	DBI_1.1.2	colorspace_2.0-3	withr_2.5.0
[13] tidyselect_1.1.2	bit_4.0.4	compiler_4.1.1	cli_3.2.0
[17] labeling_0.4.2	scales_1.1.1	genefilter_1.74.1	digest_0.6.29
[21] systemfonts_1.0.4	stringr_1.4.0	yulab.utils_0.0.4	XVector_0.32.0
[25] pkgconfig_2.0.3	fastmap_1.1.0	rlang_1.0.2	rstudioapi_0.13
[29] RSQLite_2.2.10	farver_2.1.0	gridGraphics_0.5-1	generics_0.1.2
[33] vroom_1.5.7	zip_2.2.0	RCurl_1.98-1.6	magrittr_2.0.2
[37] GenomeInfoDbData_1.2.6	Matrix_1.4-0	Rcpp_1.0.8.2	munsell_0.5.0
[41] S4Vectors_0.30.2	fansi_1.0.2	lifecycle_1.0.1	stringi_1.7.6
[45] zlibbioc_1.38.0	grid_4.1.1	blob_1.2.2	ggrepel_0.9.1
[49] crayon_1.5.0	lattice_0.20-45	Biostrings_2.60.2	splines_4.1.1
[53] annotate_1.70.0	hms_1.1.1	KEGGREST_1.32.0	pillar_1.7.0
[57] codetools_0.2-18	stats4_4.1.1	XML_3.99-0.9	glue_1.6.2
[61] data.table_1.14.2	png_0.1-7	vctrs_0.3.8	tzdb_0.2.0
[65] gtable_0.3.0	purrr_0.3.4	assertthat_0.2.1	cachem_1.0.6
[69] xtable_1.8-4	survival_3.3-1	tibble_3.1.6	AnnotationDbi_1.54.1

For manuscripts utilizing custom algorithms or software that are central to the research but not yet described in published literature, software must be made available to editors and reviewers. We strongly encourage code deposition in a community repository (e.g. GitHub). See the Nature Portfolio guidelines for submitting code & software for further information.

Data

Policy information about availability of data

All manuscripts must include a data availability statement. This statement should provide the following information, where applicable:

- Accession codes, unique identifiers, or web links for publicly available datasets
- A description of any restrictions on data availability
- For clinical datasets or third party data, please ensure that the statement adheres to our policy

The raw data, libraries, analysis log files, and analysis output files data are available under restricted access for medical data protection reasons at the European Genome-phenome Archive (https://ega-archive.org EGAD00010002223). Access can be obtained via a data access agreement. The data access agreement for this dataset corresponds to the "Harmonised Data Access Agreement (hDAA) for Controlled Access Data" as brought forward by the "European standardization framework for data integration and data-driven in silico models for personalized medicine – EU-STANDS4PM". Please contact the corresponding author for access oliver.schilling@uniklinik-freiburg.de. Requests will be answered within two weeks.
Source data, including the processed protein and peptide intensity data as well as the benchmarking results and data characteristics, are provided with this paper at Zenodo (10.5281/zenodo.6379087).

Field-specific reporting

Please select the one below that is the best fit for your research. If you are not sure, read the appropriate sections before making your selection.

☒ Life sciences ☐ Behavioural & social sciences ☐ Ecological, evolutionary & environmental sciences

For a reference copy of the document with all sections, see nature.com/documents/nr-reporting-summary-flat.pdf

Life sciences study design

All studies must disclose on these points even when the disclosure is negative.

Sample size	4 x 23 human lymph node protein samples to which defined amounts of E. coli proteins have been spiked to. Sample size was limited by the availability of FFPE tissues. For this benchmark study, this number was sufficient to achieve good separation between E.coli and human protein abundances as is shown in the manuscript.
Data exclusions	Due to unusually low identification numbers and a protein intensity distribution that was identified as an outlier, sample 28 of spike-in condition 1:6 it was not included in some visualizations. This has been noted in the Figure texts.
Replication	Replication of statistical tests was performed by bootstrapping. This leads to a robust estimate of variables. In total over 3 million independent statistical analyses were performed to asses the capability of different workflows to detect differentially abundant proteins.
Randomization	Patients were assigned to spike-in conditions in a randomized manner. The (random) date of biopsy was taken and patients were always assigned in the following manner :<br>first patient was allocated to the first spike-in condition,<br>second patient to second spike-in condition,<br>third patient to third spike-in condition,<br>fourth patient to fourth spike-in condition,<br>fifth patient was assigned to first spike-in condition and so on.<br>Additionally, block randomization was performed during the mass spectrometry data acquisition

Blinding	Blinding was not possible during data acquisition, as the ground truth of human-only samples needed to be preserved for assessing false discovery rates. As slight carry over of E.coli proteins can occur during LC-MS/MS, human-only samples needed to be measured first in each measurement batch (block).<br>During data analysis, blinding was not possible, as the generation of ROC and AUC are relying on knowing the ground truth of the dataset. For a benchmarking study employing ground-truth information, a non-blinded approach is imperative.

Reporting for specific materials, systems and methods

We require information from authors about some types of materials, experimental systems and methods used in many studies. Here, indicate whether each material, system or method listed is relevant to your study. If you are not sure if a list item applies to your research, read the appropriate section before selecting a response.

Materials & experimental systems

n/a	Involved in the study
☒	☐ Antibodies
☒	☐ Eukaryotic cell lines
☒	☐ Palaeontology and archaeology
☒	☐ Animals and other organisms
☐	☒ Human research participants
☒	☐ Clinical data
☒	☐ Dual use research of concern

Methods

n/a	Involved in the study
☒	☐ ChIP-seq
☒	☐ Flow cytometry
☒	☐ MRI-based neuroimaging

Human research participants

Policy information about studies involving human research participants

Population characteristics	From (male) patients with acinary prostate cancer histologically non-infiltrated lymph nodes were collected as sentinel samples and preserved as FFPE tissue. Age 49 to 81 years (median 65). Written consent was obtained from each patient. Detailed information of disease states in a anonymized manner can be obtained from EGA.
Recruitment	The FFPE archive of the University Medical Center Freiburg was searched for samples comprising inter-patient heterogeneity, which at the same time do not vastly vary due to different disease states (e.g cancer cohorts often suffer from varying degrees of invasiveness). We therefore chose non-infiltrated sentinel lymph nodes, which were additionally randomized for our spike-in experiments. No further selection processes were applied, as the randomized allocation of patients into different experimental groups and bootstrapping during data analysis should avoid any confounding effects (as these should be distributed equally among the different spike-in conditions. Written consent was obtained from each patient.
Ethics oversight	The study has been approved by the Ethics Board of the University Medical Center Freiburg (approval 280/18). We comply with all relevant regulations. The study was conducted in accordance to the criteria set by the Declaration of Helsinki.

Note that full information on the approval of the study protocol must also be provided in the manuscript.
